# mibPOPdb: An online database for microbial biodegradation of persistent organic pollutants

**DOI:** 10.1002/imt2.45

**Published:** 2022-08-17

**Authors:** Tanyaradzwa R. Ngara, Peiji Zeng, Houjin Zhang

**Affiliations:** ^1^ Department of Biotechnology, College of Life Science and Technology, MOE KEY Laboratory of Molecular Biophysics Huazhong University of Science and Technology Wuhan China

**Keywords:** biodegradability classification, database, environmental pollution, microbial biodegradation, persistent organic pollutants

## Abstract

Microbial biodegradation of persistent organic pollutants (POPs) is an attractive, ecofriendly, and cost‐efficient clean‐up technique for reclaiming POP‐contaminated environments. In the last few decades, the number of publications documenting POP‐degrading microbes, enzymes, and experimental data sets has continuously increased, necessitating the development of a dedicated web resource that catalogs consolidated information on POP‐degrading microbes and tools to facilitate integrative analysis of POP degradation data sets. To address this knowledge gap, we developed the Microbial Biodegradation of Persistent Organic Pollutants Database (mibPOPdb) by accumulating microbial POP degradation information from the public domain and manually curating published scientific literature. Currently, in mibPOPdb, there are 9215 microbial strain entries, including 184 gene (sub)families, 100 enzymes, 48 biodegradation pathways, and 593 intermediate compounds identified in POP‐biodegradation processes, and information on 32 toxic compounds listed under the Stockholm Convention environmental treaty. Besides the standard database functionalities, which include data searching, browsing, and retrieval of database entries, we provide a suite of bioinformatics services to facilitate comparative analysis of users' own data sets against mibPOPdb entries. Additionally, we built a Graph Neural Network‐based prediction model for the biodegradability classification of chemicals. The predictive model exhibited a good biodegradability classification performance and high prediction accuracy. mibPOPdb is a free data‐sharing platform designated to promote research in microbial‐based biodegradation of POPs and fills a long‐standing gap in environmental protection research. Database URL: http://mibpop.genome-mining.cn/

## Highlights

- This database is the first manually curated data resource on microbial biodegradation of persistent organic pollutants (POPs).
- Information on 593 intermediate compounds associated with POP‐biodegradation processes was extracted from the literature.
- A Graph Neural Network‐based model was developed to predict the biodegradability of chemical entities, which would provide a valuable tool for the risk assessment of POPs.

## Graphical abstract

This study presents the Microbial Biodegradation of Persistent Organic Pollutants Database (mibPOPdb) database, a web‐accessible literature‐based microbial biodegradation of persistent organic pollutants (POPs) resource. We also developed a robust chemical biodegradability prediction model with Graph Neural Networks. By providing high‐level curated information on POP‐degrading microbial communities, mibPOPdb will be an essential platform for fostering studies on microbial biodegradation of POP compounds and how these microbes would help solve the problem of POP accumulation. In addition, the in silico model can be used to evaluate the persistence of organic chemicals, which is a critical task in ecological risk assessment studies.

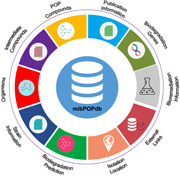

## INTRODUCTION

Persistent organic pollutants (POPs) are highly toxic and recalcitrant organic compounds that bioaccumulate through the food web and persist in the environment for extended periods [1]. These pollutants possess the potential for mobilization through the soil, water, atmosphere, and migratory species, resulting in them being widespread globally [2]. Chronic exposure to POPs has been implicated in detrimental effects on the biosphere and health [3]. The Stockholm Convention is a global treaty for the regulation of POPs, which entered into force in 2004 to protect human health and the environment [4]. Countries that are signatories to the Stockholm Convention have banned or severely restricted the use and production of POPs in the past two decades [5].

Despite the phasing out of most POPs‐based products, there is a growing body of evidence that global climate change results in legacy POP revolatilization and remobilization from surface reservoirs (e.g., permafrost, soil, water, and ice), which act as secondary sources of POPs release into the biosphere [2, 6, 7]. In addition, changes in land use and glyphosate‐induced soil erosion have reportedly resulted in the re‐emergence of legacy POPs [8, 9, 10, 11]. The resurrection of legacy POPs can potentially induce a second toxic event, which undermines global efforts to minimize human and environmental exposure to these harmful compounds [12]. As such, calls for eliminating POPs have intensified in recent years.

Employing microbial communities in the biodegradation of POPs is a relatively sustainable and ecofriendly approach to reclaiming POP‐polluted environments [13, 14, 15]. Rapid advances in high‐throughput multiomics techniques, molecular biology, bioinformatics, and relatively low‐cost next‐generation sequencing technologies have enhanced our understanding of microbial‐mediated bioremediation [16]. These advances have opened up avenues for using both culture‐dependent and ‐independent approaches in the characterization of POP‐degrading microbial communities [17]. The selection of novel microbial species and catabolic genes for the bioremediation of POPs is an important research priority [18].

There are large public data sources, such as GenBank [19], KEGG [20], and UniProtKB [21], which contain huge amounts of nucleotide and protein sequences data generated from scientific studies. However, the sheer size of the biological information data collected in these databases and insufficient annotations make it arduous to retrieve microbial biodegradation data sets from tens of millions of sequences. Hence, researchers have started developing tailored databases for specific topics to promote and facilitate easy and quick data searching and retrieval and provide tools for other researchers to analyze their own data. In this regard, the development of specialized data repositories for the organization of biodegradation of environmental pollutants data sets (i.e., curated and peer‐reviewed organisms, genes, degradation reactions, pathway maps, and publications) have greatly facilitated systems biology studies into bioremediation [22, 23], biodegradation research [24, 25], and modeling experiments [26, 27, 28] in the development of novel environmental clean‐up solutions. Several databases, accessible as web resources, have been dedicated to microbial‐mediated bioremediation of xenobiotic compounds in the last three decades. The EAWAG Biocatalysis/Biodegradation Database (EAWAG‐BBD) is an authoritative and comprehensive data repository containing biodegradation information on almost 1400 xenobiotic compounds, over 200 pathway maps, 1500 reactions, nearly 1000 enzymes, 543 microorganism entries, and 249 biotransformation rules derived from degradation reactions information extracted from scientific publications [29]. Another web resource was MetaRouter, an integrated platform that contained data on the biochemical aspects of xenobiotic compounds' biodegradation and provided tools for querying biodegradative pathways to predict a compound's biodegradability [30]. The OxDBase web resources provide information on over 240 biodegradative oxygenases extracted from scientific literature and databases and are helpful for aromatic hydrocarbons biodegradation studies [31]. The Bionemo database was a comprehensive web resource containing sequence information for over 320 biodegradation reactions, over 130 biodegradation pathways, more than 1107 proteins, and transcription regulation information of over 200 transcription units, 100 transcription factors, and 100 promoters, which was manually curated from scientific literature [32].

However, several of the abovementioned tailored web resources are no longer maintained or accessible. Furthermore, microbial‐mediated biodegradation of POPs research data is distributed randomly and unsystematically across scientific literature and public repositories, making it challenging and time‐consuming for researchers to retrieve relevant research data sets to support their own biodegradation studies without collecting unconnected and unrelated information. So far, no single dedicated web resource organizes microbial biodegradation of POPs information and provides tools for researchers to analyze their own data. Hence, it is desirable to establish a web resource of systematically reviewed microbial POP degradation information to allow for more efficient access to POP‐biodegradation data sets and facilitate data analysis and data mining, which would not be possible with experimental data stored in scientific literature.

The development of new chemicals plays a pivotal role in technological and scientific advancements and also presents serious health and environmental concerns. Chemical substances are screened to determine their persistence by assessing their ready biodegradability [33]. The bulk of chemical persistence/biodegradation evaluation studies currently uses animal‐based assays to generate evidence‐based risk profiles and develop effective risk management strategies to protect humans and the environment [34]. However, these tests are time‐consuming, expensive, and problematic from an ethical perspective [35]. Regulators advocate for the use of alternative approaches, such as in silico models, which can reliably predict the ready biodegradability of chemical substances at a reduced monetary cost and time, with the potential to reduce the number of tests on animals [33].

In recent years, several quantitative structure‐activity relationships (QSARs) classification models have been developed for predicting the ready biodegradability of chemical compounds [36, 37, 38, 39, 40]. QSAR methods build correlations between a compound's chemical structure information (described by various molecular descriptors, such as functional groups, electronic, steric, thermodynamic, and geometric properties) and a target biological property of interest [41]. However, QSAR models are inevitably associated with drawbacks that limit their application. The reliability of QSAR models and precision of ready biodegradability results is dependent on the correct feature selection applied during QSAR modeling [42]. Also, when QSAR models are applied to chemicals outside the applicability domain for which they were developed, it results in added conservatism being incorporated and an increase in error propagation within the biodegradability classification model [43]. There is a need to develop new better‐suited biodegradability classification models, which would be helped by the emergence of deep learning methods [44].

**Figure 1:**
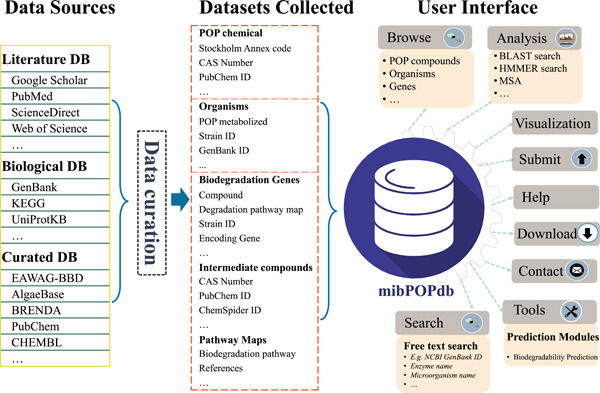
General overview of the mibPOPdb construction, content, and web interface. mibPOPdb data were collected from published literature, biological databases, and curated scientific databases, and manually curated into three main categories. The user‐friendly online interface supports data querying, browsing, uploading new data sets, and downloading various information deposited in mibPOPdb. BBD, Biocatalysis/Biodegradation Database; CAS, Chemical Abstract Service; DB, database; mibPOPdb, Microbial Biodegradation of Persistent Organic Pollutants Database; POP, persistent organic pollutant.

Herein, we report the development of a new Microbial Biodegradation of Persistent Organic Pollutants Database (mibPOPdb) that provides a centralized web server of manually curated evidence‐based microbial‐mediated biodegradation POP data sets retrieved from the scientific literature (Figure 1). The database includes information on the physicochemical properties of the POP compounds and intermediates for the breakdown reactions, experimentally verified POP degrading microbes and biodegradation genes, experimental biodegradation data, and sample collection information. To our knowledge, mibPOPdb is the first web resource that systematically provides information on microbially mediated biodegradation of POPs through a web interface, facilitating ease of browsing, querying, visualizing, and downloading POP‐degradation information contained in the database. To overcome the limitations associated with QSAR classification models, this study also presents a tool for predicting the biodegradability classification of chemicals built using Graph Neural Networks (GNNs). The GNN‐based model achieved reliable predictions for the biodegradability classification tasks and could potentially replace QSAR models in the classification of chemicals in regulatory hazard and risk assessments. The mibPOPdb is a designated open‐access platform that may assist professional POP‐biodegradation researchers and the broader scientific community working to understand the microbial biodegradation of POP compounds and promote new avenues for future research in POP bioremediation.

## RESULTS

### Compounds regulated by the Stockholm Convention

The chemicals regulated under the Stockholm Convention are aldrin, chlordane, dicofol, 1,1,1‐trichloro‐2,2‐bis(4‐chlorophenyl)ethane, dieldrin, endrin, hexachlorobenzene, heptachlor, mirex, toxaphene, perfluorooctanoic acid (PFOA), its salts and PFOA‐related compounds, polychlorinated biphenyls (PCBs), polychlorinated dibenzo‐*p*‐dioxins (PCDDs), polychlorinated dibenzofurans (PCDF), *α*‐hexachlorocyclohexane, beta hexachlorocyclohexane, chlordecone, hexabromobiphenyl, hexabromocyclododecane, hexabromodiphenyl ether, heptabromodiphenyl ether, lindane, pentachlorobenzene, pentachlorophenol and its salts and esters, perfluorooctane sulfonic acid, its salts and perfluorooctane sulfonyl fluoride, polychlorinated naphthalenes (PCNs), technical endosulfan and its related isomers, tetrabromodiphenyl ether, pentabromodiphenyl ether, decabromodiphenyl ether (c‐decaBDE), short‐chain chlorinated paraffins, and hexachlorobutadiene.

### Literature search results

Study screening and selection procedures are illustrated in Supporting Information Figure S1. The initial literature search yielded 7159 references. A total of 2486 duplicate records were detected and removed. The remaining 4673 references were eligible to be screened on the basis of the title or abstract. From these, 1986 references were considered to be ineligible and were removed. The full texts for the remaining 2687 references were manually screened, and 1623 references were excluded as they did not meet our eligibility criteria. The remaining 1064 studies were included for data extraction.

### Data content and statistics

The current version of mibPOPdb contains information on 9215 microbial strain entries annotated from 1064 peer‐reviewed articles, 184 gene (sub)families, 100 enzymes, 48 biodegradation pathways, 593 intermediate compounds identified in the biodegradation of POPs, and information on 32 toxic compounds currently targeted by the Stockholm Convention on Persistent Organic Pollutants environmental treaty. Some of the xenobiotic chemicals listed under the Stockholm Convention, such as PCBs, polybrominated diphenyl ethers (PBDEs), PCNs, PCDDs, and PCDFs are not single compounds but rather occur as complex mixtures of congeners. Although there are hundreds of PCBs, PBDEs, PCNs, PCDDs, and PCDFs, only a small group of the congeners exhibit great toxic potential [5]. The chlorination/bromination pattern of these compounds determines their level of toxicity [45]. The congeners with a coplanar structure exhibit the most toxicological effects based on combined health effects considerations [46]. For each group of a mixture of compounds, the most studied and most toxic congener was selected as a model compound to study the biodegradation process for that group of compounds. For example, for PCDDs, the most studied and toxic of them is 2,3,7,8‐tetrachlorodibenzo‐*p*‐dioxin, and in this study was selected as the model compound to study the biodegradation of PCDDs.

**Figure 2:**
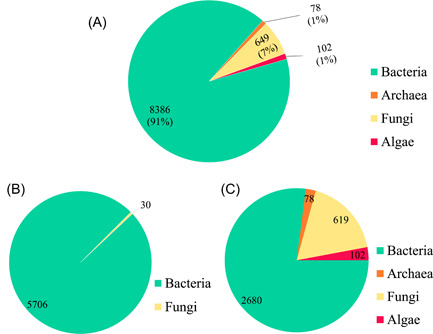
Statistics of the collected POP‐degrading microbial data. (A) Overall distribution of microorganisms in mibPOPdb. (B) Distribution of POP‐degrading microbial strains collected from literature based on functional gene analysis. (C) Distribution of POP‐degrading microbes collected from literature based on either phylogenetic or functional screening studies. POP, persistent organic pollutant.

More than 90% of the microbial data set comprises bacterial entries, followed in order of abundance by fungal, algal, and archaeal microbial entries (Figure 2A). The POP‐biodegradation functional gene data set in mibPOPdb is composed of 5736 microbial strain entries (Figure 2B). Of these entries, 5706 bacterial strains and 30 fungal strains accounted for 99.48% and 0.52%, respectively, of the POP‐biodegradation functional genes data set in mibPOPdb (Figure 2B). The mibPOPdb contains a total of 184 POP‐related biodegradation gene (sub)families covering 22 of the 32 POP compounds currently listed under the Stockholm Convention (see Supplementary File: mibPOPdb data.xlsx). There are 10 POP compounds for which no genes from any organism have been linked to their degradation (Supporting Information Table S1). Strain level information in mibPOPdb was assigned by manually annotating data from species descriptions in the primary literature based on small subunit ribosomal RNA (16S/18S rRNA) and genome sequence information. The mibPOPdb database has information on 3479 microbial strain entries collected from scientific literature, with 77.03% comprising 2680 bacterial strains entries, with fungi, algae, and archaea, accounting for 17.79%, 2.93%, and 2.24%, respectively (Figure 2C).

### Prediction model training setting and performance analysis

The statistical parameters adopted for evaluating the classification performance of the five models were sensitivity (Sn), specificity (Sp), balanced accuracy (BA), and error rate (ER). This is a two‐class model (i.e., readily biodegradable [RB] and not readily biodegradable [NRB]), considering that the specificity of one class corresponds to the sensitivity of the other class. These statistical parameters are all taken into account when evaluating the predictive ability of a model because they help describe the behavior of the model, for example, avoiding RB molecules being classified as NRB (which is associated with high NRB specificity) or avoiding NRB molecules being classified as RB (related to high NRB sensitivity) [47].

The individual classification performance for fivefold cross‐validation on the training set and external validation set for the five GNN models is collected in Table 1. The sensitivity and specificity of NRB class, BA, and ER for each GNN model are reported for the training and external validation sets. All five proposed classification models displayed a high average performance with BA values ranging between 0.89–0.93 and 0.88–0.90 for the final test and external validation data sets, respectively. From the cross‐validation results, model 4 exhibited the highest average performance for both the final test and external validation data sets, with BA values of 0.93 and 0.90, respectively. The ER of model 4 was the lowest among the five models, with 0.07 and 0.10 ER values for the final test and external validation data set, respectively. All the proposed classification models demonstrated the same trend in results, that is, the specificity (Sp) is higher than sensitivity (Sn), implying that the models can correctly classify NRB molecules with a more stable prediction capability compared to RB molecules. The specificity, sensitivity, BA, and ER of the values obtained on the test and external validation data sets in the five proposed classification models' cross‐validation results were comparable, demonstrating the reliability and robustness of the proposed classification models. Of the five proposed GNN‐based classification models in Table 1, model 4 displayed the best performance for specificity and sensitivity on both the testing and external validation data sets and was used to predict the biodegradability of chemical molecules.

**Table 1:** Classification performance for fivefold cross‐validation on testing set and external validation data sets

Model	Final test data set				External validation data set			
	BA	Sn	Sp	ER	BA	Sn	Sp	ER
Model 1	0.91	0.87	0.95	0.09	0.88	0.82	0.94	0.12
Model 2	0.91	0.87	0.95	0.09	0.88	0.81	0.94	0.12
Model 3	0.89	0.85	0.93	0.11	0.88	0.82	0.93	0.12
Model 4	0.93	0.90	0.96	0.07	0.90	0.85	0.95	0.10
Model 5	0.91	0.87	0.95	0.09	0.89	0.81	0.96	0.11

*Note*: For each model, sensitivity (Sn, correctly predicted ready biodegradable), specificity (Sp, correctly predicted not ready biodegradable), balanced accuracy (BA, average of sensitivity and specificity), and error rate (ER, complement of balanced accuracy) are provided.

The GNN model presented in this study was compared with the QSAR models presented by Mansouri et al. [40]. The results for the final and external validation data sets in Table 2 show that the proposed GNN‐based model exhibits a relatively moderate improved classification performance compared to the QSAR models already published in the literature. The GNN model had a slightly higher average performance, with BA values of 0.93 and 0.90 for the test and external validation sets, respectively. The QSAR models had lower average performances. The partial least squares discriminant analysis model had the lowest balance accuracy value of 0.85 for the test set, and the support vector machines model had the lowest balance accuracy value of 0.82 on the external validation set. The GNN‐based model had the lowest ERs compared to the QSAR models on both the test and external validation data sets (ER values of 0.07 and 0.10, respectively). This proves that the GNN model can rapidly and accurately predict the biodegradability of molecules. The GNN model exhibits a similar trend to that observed in the QSAR models: specificity being higher than sensitivity for the final test and external validation sets. The GNN classification model predicted and classified NRB molecules more accurately than RB molecules.

**Table 2:** Comparison of classification performance between GNN model and previously published QSAR models estimated on the same biodegradability experimental data set

Model	Final test data set				External validation data set			
	BA	Sn	Sp	ER	BA	Sn	Sp	ER
*k*NN	0.86	0.81	0.90	0.14	0.83	0.75	0.91	0.17
SVM	0.87	0.82	0.91	0.13	0.82	0.74	0.91	0.18
Consensus 1	0.87	0.82	0.92	0.13	0.83	0.76	0.91	0.17
Consensus 2	0.91	0.88	0.94	0.09	0.87	0.81	0.94	0.13
PLSDA	0.85	0.83	0.87	0.15	0.83	0.80	0.86	0.17
GNN	0.93	0.90	0.96	0.07	0.90	0.85	0.95	0.10

*Note*: For each model, sensitivity (Sn, correctly predicted ready biodegradable), specificity (Sp, correctly predicted not ready biodegradable), balanced accuracy (BA, average of sensitivity and specificity), and error rate (ER, complement of balanced accuracy) are provided.

Abbreviations: GNN, Graph Neural Network; *k*NN, *k*‐nearest neighbor; PLSDA, partial least squares discriminant analysis; QSAR, quantitative structure‐activity relationship; SVM, support vector machines.

### Data access and usage

#### Web interface and data browsing

The mibPOPdb database is freely accessible through a user‐friendly website (http://mibpopgenome-mining.cn) and offers biological researchers access to information on microbially mediated biodegradation of POPs. Through a user‐friendly interface, mibPOPdb provides tools for browsing, querying, exploring detailed information on microbial degradation of POPs, downloading all data, and a series of online bioinformatics services and a chemical biodegradability prediction tool (see Figure 1).

**Figure 3:**
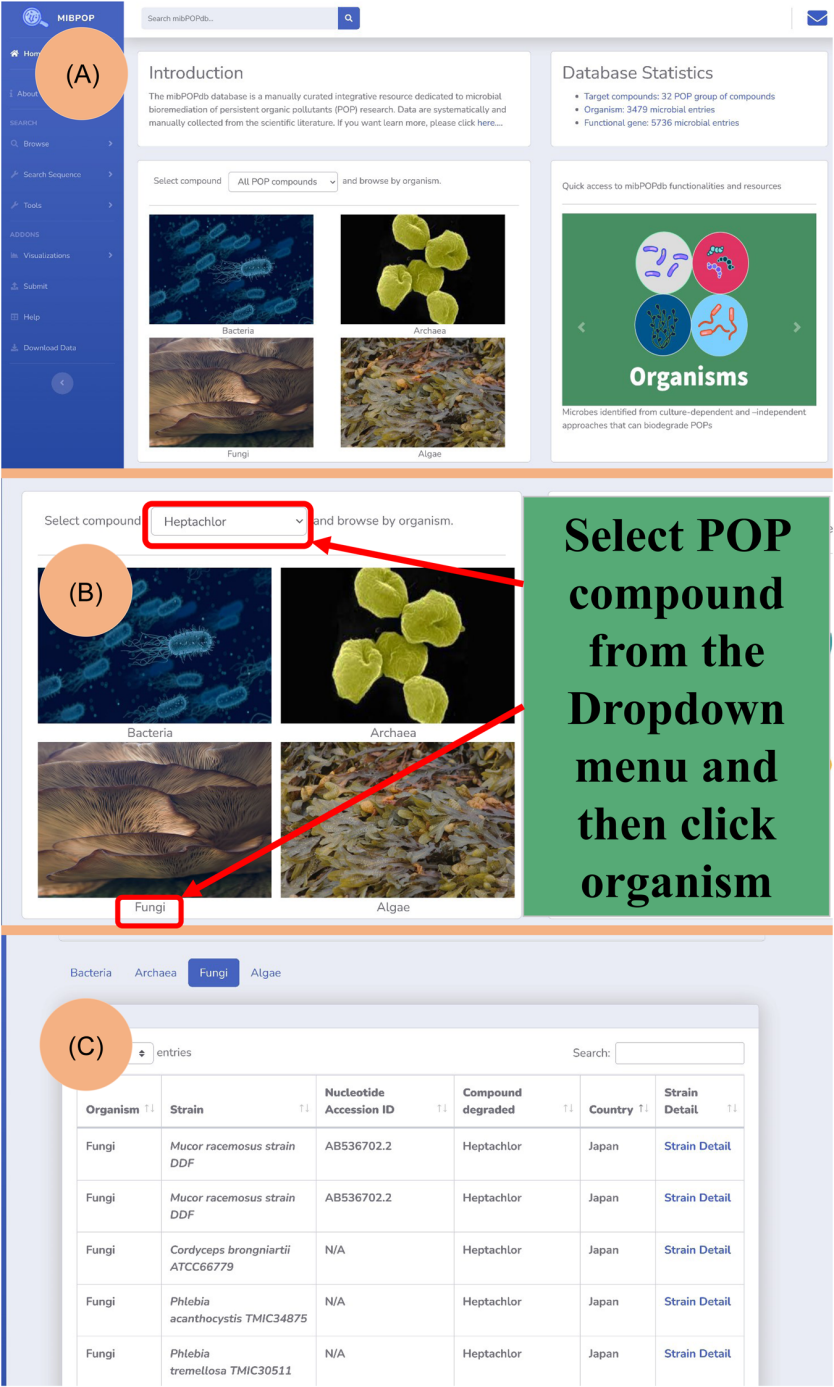
User interface of the mibPOPdb. (A) The mibPOPdb homepage. (B) Dropdown menu. Users can quickly select their POP compound from the list and organism of interest and retrieve its microbial POP degradation data sets. (C) The quick data browsing results using the dropdown menu services on the homepage. mibPOPdb, Microbial Biodegradation of Persistent Organic Pollutants Database; POP, persistent organic pollutant.

The homepage user interface is very simple (Figure 3A). From the mibPOPdb home page, users can quickly access and retrieve microbial biodegradation data sets of a specific POP compound by choosing the POP compound from the list of POP compounds in the dropdown menu and then selecting the type of microorganism for which they want data sets to be shown (Figure 3B).

Their query will be returned displaying organisms capable of degrading that POP compound (Figure 3C). The “Tools” page contains the chemical biodegradability prediction tool. The “About Us” and “Help” pages display information about navigating the database. The “Search sequence” page provides a suite of online bioinformatics services for users to execute sequence comparative analysis studies within the framework of the database.

The “Browse” page has six subpages, that is, compounds, organisms, biodegradation genes, intermediate profiles, degradation pathway maps, and enzymes. Users can find the basic annotation of the following:

“Compounds” page, such as POP compound name, listing year, Chemical Abstract Service (CAS) number, European Community number, and DSSTOX substance ID. Clicking on the “POP details” button directs the user to the report card page containing detailed information for that specific compound. The information presented on this page comprises the compound's listing information, general descriptions of the compound, structural analogs, and publication information. Users can backtrack to literature sources offering reports for that compound. Also provided are links to external resources, such as ChemSpider, DSSTOX, PubChem, and European Chemicals Agency (ECHA), which also display information concerning that compound (Supporting Information Figure S2).

“Organisms” page, such as type of organism, strain ID, nucleotide sequence accession ID, compound degraded, and country from which the microbial sample was taken. Users can access the detailed information for a specific strain by clicking the strain detail button. The detailed information report card page contains the strain's general information, the location where the original environmental samples were collected, POP compound metabolized, bioremediation information, and reference to the scientific literature (Supporting Information Figure S3).

“Biodegradation genes” page includes the type of organism, strain ID, encoding gene, protein sequence accession ID, and compound degraded. The biodegradation gene detailed information page provides information on the compound metabolized, geographical location from where the sample was taken, link to scientific literature, and degradation gene information, such as encoding gene, enzyme name, UniProt ID, and sequence accession IDs (Supporting Information Figure S4).

“Intermediate profiles” page, such as POP degraded, intermediate compound, and the CAS number of the compound. The detailed information page for a specific intermediate compound profile can be accessed by clicking on the intermediate detail link. The detailed report card contains information on the POP degraded, POP degradation pathway, the intermediate compound identified, and the intermediate compound's physicochemical properties. The PubChem, KEGG, and ChemSpider IDs are provided as external links (Supporting Information Figure S5).

The “Pathway maps” page contains a dropdown menu of the POP degradation pathways constructed and drawn relying on literature reporting microbial POP degradation that has been proven experimentally. The detailed report card for each biodegradation pathway displays information on the compound degraded, a general description of the POP compound, a graphical display of the POP compound's biodegradation routes, and literature citations so that users can backtrack to the original scientific research reporting the biodegradation routes (Supporting Information Figure S6).

And the “Enzymes” page, users can access information about the general description of enzyme function, enzyme class, enzyme classification number, enzyme name, and its synonyms, degradation pathway/s that they are associated with, reactions catalyzed by the enzyme, encoding gene and gene clusters, external links to BRENDA, KEGG, ExPASy, and Enzyme Database, microorganism information, links to GenBank, protein ID, and UniProtKB, and literature citations (Supporting Information Figure S7).

Moreover, when browsing entries in the compounds, organisms, biodegradation genes, and intermediate profile pages, users can use the interactive free‐filter tabs to show/display a data set range based on their own chosen criteria. Free‐text filters help users focus on specific information and perform efficient data analysis according to their set criteria. In addition, for ease of browsing, an interactive navigation bar is implemented at the bottom of each detailed information page aiding users in traversing the different sections of the detailed page quickly at the click of a button.

#### Data query

**Figure 4:**
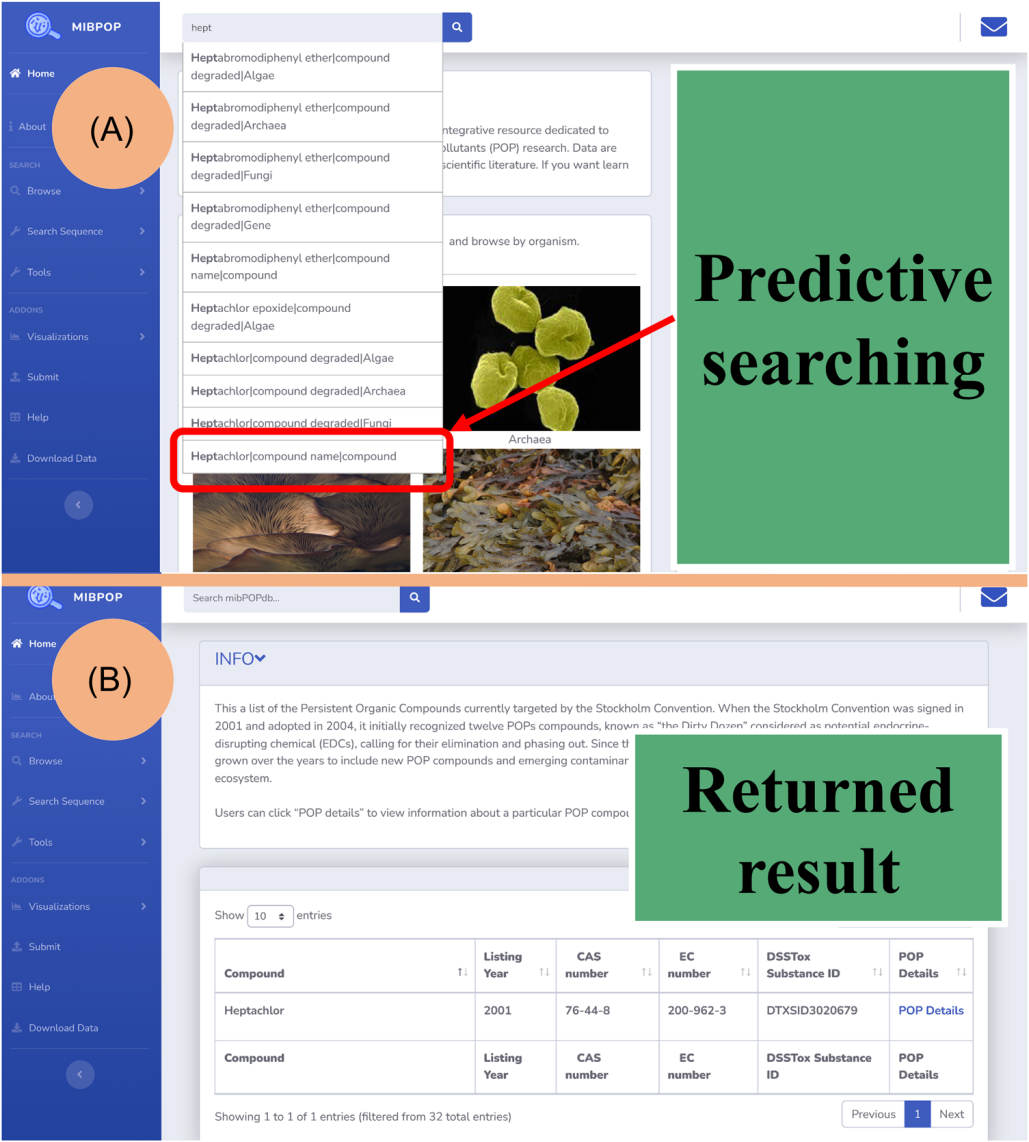
mibPOPdb data querying. Users can query mibPOPdb data through four paths: “Search by compound,” “Search by CAS ID,” “Search by protein and nucleotide sequence accession number,” and “Search by compound degraded.” Free texting and predictive searching are supported. (A) The search interface of mibPOPdb with compound name as the input. (B) The query's result based on “Search by the compound name” returns information on the POP compound Heptachlor. CAS, Chemical Abstract Service; mibPOPdb, Microbial Biodegradation of Persistent Organic Pollutants Database; POP, persistent organic pollutant.

Using the simple search bar available in the upper left corner of each webpage, users can search the database for POP compounds, intermediate compounds, POP degradation genes, or POP degrading microbes of interest. Free text and predictive searching are supported, facilitating more straightforward and faster information searching in mibPOPdb. Users can query mibPOPdb through four paths: “Search by the compound name,” “Search by CAS ID,” “Search by protein and nucleotide sequence accession number,” and “Search by compound degraded.” The user can input a few characters of the prefix word of what they want to search in the search bar. Possible suggestions based on data found in mibPOPdb will be displayed in the format “value|data field|data table.” Value refers to data entry in mibPOPdb with the same prefix as the user's input, and the data field is the location where the value is stored. The data table is the domain that the user might want the search to focus on in the context of the database, for example, “Heptachlor|compound name|compound.” Heptachlor is a compound name, and users can search for its information in the compound domain (Figure 4A,B). In addition, the mibPOPdb provides a series of bioinformatics utilities for sequence analysis on the “Search sequence” page, including BLAST, Clustal Omega, and Phylotree modules (Supporting Information Figure S8). One can use the BLAST sequence similarity search to find a POP‐degrading microbe or its homologs. The Phylotree.js module implemented in mibPOPdb facilitates studies into the evolutionary relationships between the user's query sequence and local sequences in the mibPOPdb database.

**Figure 5:**
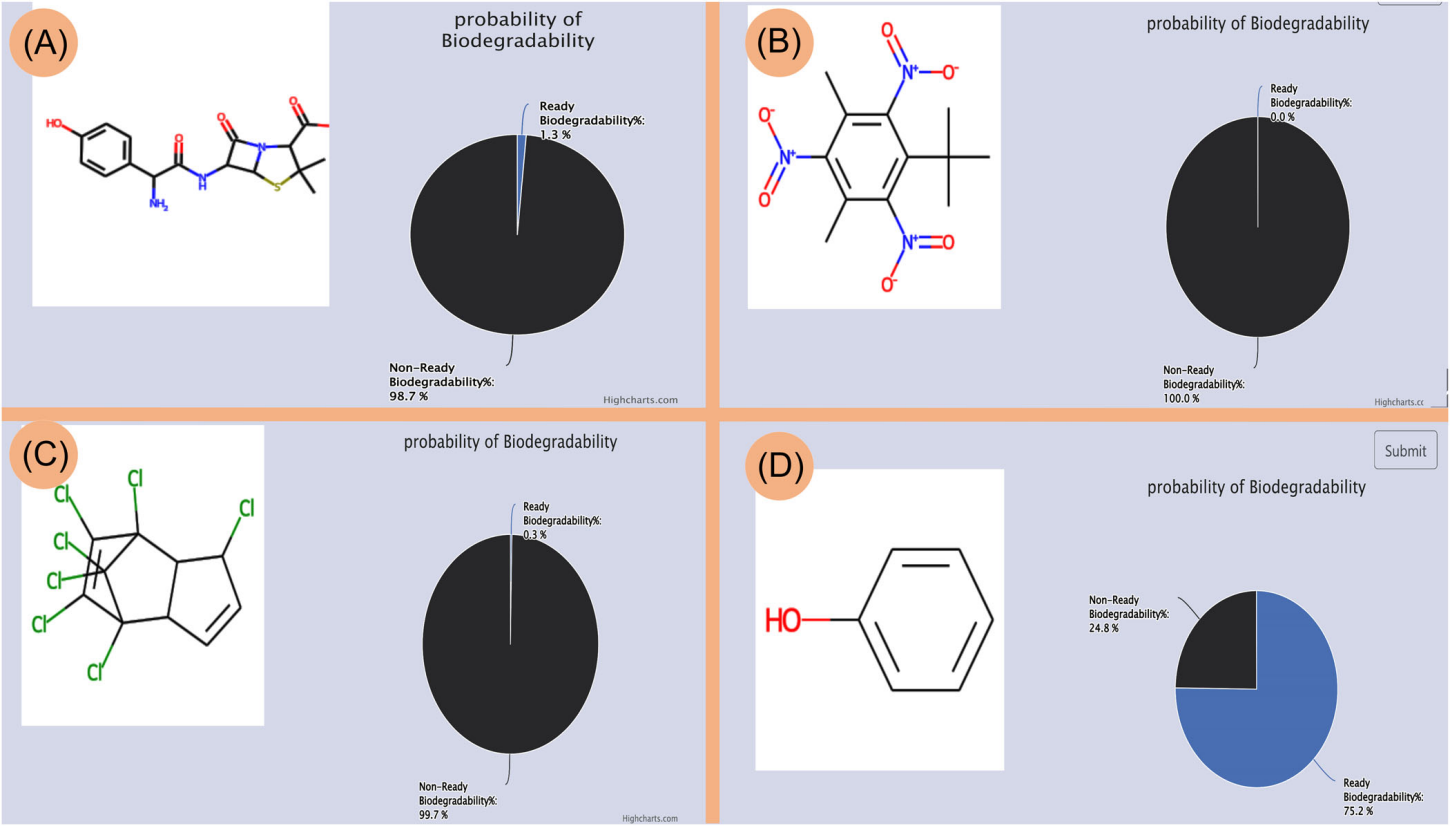
Predicting the biodegradability of chemical compounds. Users can enter the SMILES string for their query compound. The results window shows the biodegradability probability for four representative compounds. (A) Amoxicillin, (B) musk xylene, (C) heptachlor, and (D) phenol.

The mibPOPdb provides a tool for predicting the ready biodegradability of chemical compounds. Users can input the SMILES string of any arbitrary single chemical compound (this includes compounds not covered in the Stockholm Convention list) to determine its ready biodegradability. The application range of the prediction tool is limited to predicting the ready biodegradability of organic compounds. In addition, there is a limitation associated with the tool's applicability when predicting the ready biodegradability of complex or a mixture of compounds with undefined structures, such as pentabrominated diphenyl ether technical mix (DE‐71), which does not have a SMILES string value assigned to it. Users are encouraged to submit SMILES of a specific chemical compound to predict the chemical biodegradability as SMILES cannot be obtained for mixtures of compounds. An example of the tool's chemical biodegradability prediction results for the compounds amoxicillin, musk xylene, heptachlor, and phenol is illustrated in Figure 5.

The output returns a pictorial graphic of the structure of the compound and the predicted biodegradation probability value as a percentage. As mentioned earlier, based on the Japanese Ministry of International Trade (MITI) (1) [48] biodegradability screening test, a compound is described as RB if the predicted ready biodegradability value is equal to or greater than 60%. If the predicted ready biodegradability value is less than 60%, the compound is classified as NRB. Antibiotics like amoxicillin are not easily biodegradable or nonbiodegradable. In this study, amoxicillin had a predicted nonready biodegradability value of 98.7% (Figure 5A). Musk xylene, a well‐known nonreadily biodegrade compound [49], was predicted to be 100% nonready biodegradable (Figure 5B). Heptachlor is a POP compound and is NRB [50]. Heptachlor had a nonready biodegradability value of 99.7% (Figure 5C). The compound phenol is a benchmark chemical in screening tests and is used as a ready biodegradable standard reference [49], and its predicted ready biodegradability value was 75.2% (Figure 5D).

### Data visualization

Interactive data visualizations help users to better probe and understand the POP‐degrading data in the mibPOPdb. The interactive pie chart maps display the statistical information for microbial biodegradation of POP studies based on where the original environmental samples were collected. The interactive map can also help show users by continents and areas where data sets for microbial degradation of POPs studies are still to be undertaken or missing. By clicking on any given data point on the map, one immediately accesses the POP‐biodegradation data sets associated with that geographical location. Hence, the data visualization page offers the ideal starting point for exploring the data in mibPOPdb.

### Additional functionalities of the database

*Submission of data*: mibPOPdb incorporates an interactive data submission feature to encourage contributors to help complement the web resource by submitting newly published data sets related to microbial degradation of POPs following the devised submission guidelines. The curator may engage the researchers who have submitted novel data sets to review any potentially missing data during the information validation stage. It will then be uploaded and integrated into the database.

*Downloading data*: All data sets used in constructing the mibPOPdb can be freely accessed on the download page. Users are strongly encouraged to cite the original works when redistributing or reproducing the data sets.

## DISCUSSION

Despite the establishment of specialized web resources dedicated to microbial‐mediated biodegradation of xenobiotic compounds during the last 30 years, several of them have either gone offline or are no longer maintained. In addition, the systematic collection of validated POP degrading microbes is rare. Successful microbial‐based biodegradation of POPs hinges on the characterization of microbial communities from diverse environments to investigate their ecology and biodiversity and determine their POP bioremediation capabilities [51]. Thus, establishing an integrated data repository that contains genomics, proteomics, and results of biodegradation experiment research data for POP‐degrading microbes is an important development.

We built mibPOPdb, a manually curated and open‐access data resource that provides information on experimentally validated POP‐degrading microbial communities. The mibPOPdb database contains information on POP compounds listed under the Stockholm Convention, POP‐degradation associated functional genes and strains, intermediate compounds of the degradation processes, and results of POP‐biodegradation experimental studies.

Bacteria have been the foci for bioremediation studies; however, in recent times, archaea and eukaryotes have been shown to play an important role in the biodegradation of xenobiotic compounds [52, 53]. The availability of mibPOPdb, an integrated web resource displaying information on a wide variety of POP‐degrading algal, archaeal, bacterial, and fungal strains, can facilitate the development of novel POP bioremediation approaches that draw on the abilities of underutilized microbial domains. Currently, the role of archaea in the degradation of POPs remains unclear. Studies have revealed that methanogens play a key role in the anaerobic biodegradation of chlorinated pollutants by cometabolic processes [54]. Reductive dechlorination of POPs by fermentative bacteria under methanogenic conditions is accompanied by the production of acetate or hydrogen as waste products [55]. Methanogens are capable of reducing the concentration of acetate and hydrogen, which increases the thermodynamic favorability and drives forward the anaerobic degradation processes [54]. In addition, organohalide‐respiring bacteria lack the ability to synthesize corrinoids, which are enzyme cofactors essential in the functionality of reductive dehalogenase systems [56]. In these anaerobic environments, archaea may provide the key corrinoids for these dechlorinators [57].

By incorporating manually curated information on POP‐degrading microorganisms beyond the typical bacterial candidates, mibPOPdb can build the foundation for more studies into the discovery of novel POP‐degrading enzymes and lead to the development of new POP bioremediation strategies. In addition, mibPOPdb provides experimentally validated data sets on the intermediary metabolites and end‐products produced during POP compounds biodegradation. This information is vital in helping elucidate POP degradation pathways [58].

The sharing, availability, and reuse of POP‐biodegradation experimental data can help accelerate the development of POP‐biodegradation research [59]. Despite the availability of experimentally validated POP‐biodegradation results, they are usually reported in scientific literature and are hard to mine, limiting the visibility and availability of experimental data in POP degradation research studies [17]. The mibPOPdb aims to fill the gap by improving the accessibility of these POP degradation experimental data sets by integrating the manually curated experimental details into the data repository. The development of novel technologies for efficiently removing POPs and the intermediary metabolites of their breakdown processes from the environment depends on consolidating such data sets.

Computational‐based approaches are gradually becoming important in predicting and evaluating the ready biodegradability of chemical substances [36]. Several QSAR classification models are used to predict the ready biodegradability of chemicals. However, the complex implementation of QSAR models limits their functionality [60]. GNNs have been successfully used in various biological fields that process graph structure data, such as molecular activity and property, synthesis, and interaction predictions [61]. Under the molecular graph theory, molecular structures can be interpreted as a chemical graph, where a molecule's atoms and bonds are mapped into sets of nodes and edges, respectively [62]. This type of representation is useful as an input feature in graph studies, allowing for the mathematical processing of molecular structures [61]. Features are automatically extracted from raw inputs, whereas QSAR classification models are influenced by some degree of bias because the selected handcrafted features or predefined descriptors might leave out important structure information [63].

The GNN model developed in this study exploited the features of atoms in the molecular graphs to predict the ready biodegradability of a chemical compound and achieved a higher overall classification performance compared to that of QSAR models reported in published literature. In addition, the GNN model displayed a stable classification performance because it does not make use of predefined molecular fingerprints as in the case of traditional machine learning models, which require performing complex feature selection processes and have complex interpretations [63]. Understanding the structure of a chemical is important in the chemistry field [64]. The RDKit generates pictorial depictions of the query compounds as images. Determining the structural formula of the query compound provides scientists with a visual representation of its chemical formula. In cheminformatics, chemical images are combined with deep learning algorithms for predicting chemical toxicity without employing any chemical descriptors [65].

## CONCLUSIONS

This study presents the mibPOPdb, a manually curated data repository that focuses on the microbial degradation information of POPs extracted from the scientific literature. One of the limitations of this study, however, is that only English language reports were retrieved. The mibPOPdb portal also incorporates a GNN‐based prediction module, which can be used to assess and predict the ready biodegradability of chemical substances. The mibPOPdb database is an ideal central point for scientists looking for specialized information on microbial degradation of POPs, which can be used to foster research into POP degrading microbial communities and the development of efficient POP bioremediation strategies.

## METHODS

### General approach to database construction

The construction of the mibPOPdb database was as follows: briefly, specific literature search strategies were developed and included broad search terms derived from the main concepts of this study work to collect validated data associated with microbial biodegradation of POPs. Publicly accessible scientific literature databases were then searched to retrieve all relevant articles reporting microbial POP degradation that has been proven experimentally. Eligible studies were identified by manually screening the titles and abstracts of this article pool. Studies that were clearly not related to the microbial degradation of POP compounds listed under the Stockholm Convention or the intermediate compounds obtained in the breakdown pathways of the POP degradation were removed at this stage. Full texts for the studies were then obtained and manually screened the files using the inclusion and exclusion criteria (Supporting Information Table S2). This was done to ensure the high quality of our data and to remain with the primary literature. This was followed by manual curation of experimentally supported events. Lastly, the chemical biodegradability module and the website services were implemented. A summarized graphical description of the mibPOPdb database content and construction is outlined in Figure 1.

### Data collection and processing

To find the validated data associated with the microbial degradation of POPs, the Web of Science, Google Scholar, ScienceDirect, and PubMed literature databases were systematically searched to retrieve relevant scientific literature. A combination of Medical Subject Heading terms with a list of keywords, such as microbial degradation, biometabolism, biomineralization, biotransformation, decomposition, microbial bioremediation, catabolism, the specific name of every compound in the Stockholm Convention list, and specific name/s of the intermediate metabolites identified in the breakdown pathway was used to perform the literature searches. The search strategy employed for the PubMed database is shown in Supporting Information Table S3 (also see Supplementary File: mibPOPdb data.xlsx for the list of POP compounds listed under the Stockholm Convention and the intermediate compounds identified during their biodegradation). Over 7000 indexed citations were retrieved as a result of the search and imported the search results into EndNote (version X8.1). Manually screened all records to ensure that they had title records. After investigating the references, titles were manually added to documents with gaps in the Title field. Duplicates in the EndNote library were removed automatically in a series of steps that required changing which fields were compared by EndNote. The set field preferences for the deduplication process are shown in Supporting Information Table S4. The next stage was visually scanning the references and manually removing the duplicates that were not picked up automatically by comparing the titles of the articles. In addition, the articles were manually vetted for suitability for inclusion in this study by manually screening their titles and abstracts. At this stage, studies that were clearly not related to the microbial degradation of POPs listed under the Stockholm Convention and the intermediate compounds of their degradation and articles not published in the English language were excluded.

Furthermore, full texts for the studies were retrieved. The full texts of the studies were then manually screened using the inclusion/exclusion criteria. At this stage, systematic review articles, editorials, conference abstracts, and letters from which primary data cannot be extracted; publications that reported the degradation of the POP compounds but did not identify the microbial species responsible for the degradation of those POPs, and studies that reported the improvement or enhancement of POP‐biodegradation efficiencies using microbial species already identified in other degradation research studies for that same POP compound were excluded. Manually reviewed the full text for the primary literature to assess and ensure the high quality of our data. Two researchers (Tanyaradzwa R. Ngara) and (Peiji Zeng) were tasked with manually extracting the microbial degradation of POPs data sets from the eligible scientific literature and resolved areas of disagreement by consensus through discussion and consulting a third reviewer (Houjin Zhang). Three types of data were extracted from the eligible list of papers, that is, (i) functional genes encoding enzymes involved in the biodegradation of POPs, (ii) POP compound degraded and intermediate compounds of the biodegradation reaction, and (iii) microbial species experimentally validated to biodegrade POPs.

#### POP‐degrading organism resources

Two independent investigators (Tanyaradzwa R. Ngara and Peiji Zeng) extracted all relevant data from each included literature, reporting microbial POP degradation that has been proven experimentally. The target data fields used to capture the data extracted from the included studies are shown in Supporting Information Tables S5 and S6. All microbial entries of the mibPOPdb database are categorized into two groups, that is, organisms and biodegradation genes, contingent on identifying POP degrading microbes, either phylogenetic marker or functional gene marker. To develop a more authoritative knowledge‐base for microbial degradation of POPs, where appropriate and convenient manually curated POP‐degrading microbial information taken from the literature is linked to external resources, such as AlgaeBase [66], BRENDA [67], EAWAG‐BBD [29], GenBank [19], KEGG [20], and UniProtKB [21].

#### Collection of POP compound information and intermediate compounds formed in the breakdown pathway

Data associated with the toxic chemicals listed under the Stockholm Convention treaty and the intermediate compounds formed during the metabolism of these xenobiotic compounds were retrieved from the scientific literature. For each of the POP compounds, data such as the compound name, Stockholm annex code, year of listing decision, compound's physicochemical properties, structure, and structural analogs (i.e., chemical analogs with the highest structural similarity to the toxic parent compound list under the Stockholm Convention) were collected. Scientific literature descriptions were retrieved from PubMed. The information was categorized into four broad sections: POP listing information, Compound description, Structural analogs, and Publications. For each POP compound record in our data set, their PubChem, KEGG, CHEMBL, DSSTOX Substance, CAS number, and ECHA IDs are externally linked to the corresponding compound information in those external databases. The data field for POP compounds is shown in Supporting Information Table S7. In addition, the two investigators extracted data associated with the intermediate compounds identified during the metabolism of the xenobiotic compounds covered by the Stockholm Convention reported in the scientific literature. For each eligible intermediate compound entry in mibPOPdb, the following basic characteristics were extracted: intermediate name, POP degraded, POP degradation pathway, SMILES string, CAS number, PubChem ID, KEGG ID, and ChemSpider ID. The data field for the intermediate compound entries is shown in Supporting Information Table S8.

### Biodegradability experimental data set used to evaluate the GNN model's performance

The data set used to train the model and validate model performance was published by Mansouri et al. [40] and was composed of 1725 chemicals belonging to either of the two categories of compounds, that is, RB and NRB. It comprised training, testing, and external validation subsets (Supporting Information Figure S9). SMILES strings were used for molecular structure representation of the compounds and easy encoding of the molecular graph [68]. PubChem [69] and ChemSpider [70] were used to verify the accuracy of SMILES. The chemicals were checked for duplicates based on SMILES matching. The total number of RB and NRB compounds in the data set was 547 and 1178, respectively. The chemicals are classified into either RB or NRB based on the MITI test [48, 71]. The MITI‐I screening test evaluates the biodegradability of chemical substances by measuring the biochemical oxygen demand (BOD) in an aerobic aqueous medium over a 4‐week test period. Chemical substances that are described as RB are those with a BOD value of 60% or higher, and NRB chemicals are those considered to have a BOD value of lower than 60% [72].

### Development of a GNN model for ready biodegradability

**Figure 6:**
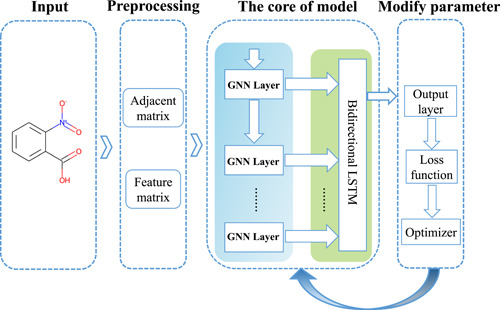
Development of GGN‐based model for predicting chemical biodegradability. It comprises three main parts: (1) Preprocessing of data, (2) molecular graph representation of the chemical using GNNs, and (3) modifying parameters of GNNs using an optimizer based on the value of the loss function. GNN, Graph Neural Network; LSTM, long short‐term memory.

The general concept of GNN is based on a recursive message passing neural network scheme [73]. In graph representation learning, the molecule's spatial structure information, node features, and edge features are taken as inputs in order for the GNN to learn the representation vector for each node of the graph. Each node in the graph aggregates features information from its neighbors to iteratively update its new representations [74]. At the last iteration, an entire graph's representation vector is obtained by pooling together the representation vectors of all the nodes in the graph [75, 76]. The pipeline for the model development is outlined in Figure 6, and it essentially involves three parts: (1) preprocessing of data, (2) using GNNs to model the representation of the chemical graph, and (3) modifying parameters of GNNs using an optimizer based on the value of the loss function.

#### Preprocessing of data

To predict the biodegradability of a chemical molecule, GNN needs to know the atom's molecular graph structure and feature vector (see Supporting Information Table S9). First, we used the RDKit software package [77] to generate a molecular graph and an adjacent matrix to represent it.

The SMILES strings were converted into molecular graphs. The molecular graph denoted as *G* = (*V*, *E*) can be described as the connectivity relations of a set of vertices (*V*), representing the nodes, and a set of edges (*E*), representing the connections between the nodes in *V*. For adjacent matrix $A^{n*n}$, n is equal to the number of the atom and the value of the component $a_{\mathrm{ij}}$ indicates a connection from node j and to node i. In this study, we define *A* by

(1) $$a_{ij}=\left\{\begin{array}{ll} 1 & \text{if}\;\text{there}\;\text{is}\;\text{an}\;\text{edge}\;\text{from}\;\text{node}\;j\;\text{to}\;i,\\ 0 & \text{otherwise}. \end{array}\right.$$

Finally, we used the canonical atom featurizer to generate the atom feature vector of the molecular graph [78].

#### Architecture of GNN

The idea of message passing is straightforward; at each iteration, each node feature will be updated through aggregate information from its local neighborhood. During message passing, each node representation will update through three functions: (1) message function, which can generate message information of the node; (2) aggregation function, for node v∈V, this function is responsible for aggregating local message information from its local neighborhood node u∈N(v); and (3) update function, it will combine aggregated information and node v self‐feature vector to update node v.

The above three functions can help each node learn spatial structure information of the graph and then use the readout function to obtain a graph representation, as shown in Supporting Information Figure S10.

We redesigned a model for predicting biodegradability according to Graph Isomorphism Network [79], a classic GNN model. Details can be expressed as follows:

(2) $$\begin{array}{l} m_{u}^{t}=\sigma \left(Wh_{u}^{t-1}\right),\;\;\forall u\in N\left(v\right),\\ h_{N\left(v\right)}^{t}=\sum_{u\in N\left(v\right)}m_{u}^{t}m_{u}^{t},\\ h_{v}^{t}=\text{GRU}\left(\text{CONCAT}\left(\left(1+\epsilon \right)*h_{v}^{\left(t-1\right)},h_{N\left(v\right)}^{t}\right)\right),\\ z_{G}^{t}=\text{WeightSumAndMax}(H^{t}), \end{array}$$

where the superscript t or t−1 indicates the layer of GNN. $h_{u}$ is the feature vector of node u, N(v) is the local neighborhoods of node v, $z_{G}$ is the representation of the graph, H is the feature matrix consisting of all of the feature vectors of the node, and ϵ is a learnable parameter or a fixed scalar whose value one can decide on during model training. The message function is a linear perceptron based on message passing and produces message information on each node. The aggregation function is the summation of local neighborhoods' message information, and the update function will feed the vector concatenating $(1+\epsilon )*h_{v}^{\left(t-1\right)}$ with $h_{N(v)}^{t}$ into gated recurrent units [80], whose result will assign node v.

Instead of generating graph representation on the last GNN layer, it will be generated after each iteration. After updating all node features, weight sum and max function will generate a graph presentation vector by concatenating weight sum and maximizing each dimension on node feature vector H. Finally, all of the graph representation $z_{G}^{i}$ will be fed into long short‐term memory (LSTM) [81] and generate the molecular fingerprint $z_{G}$, which can be expressed as follows:

(3) $$z_{G}=\text{LSTM}\left(z_{G}^{1},\text{…},z_{G}^{T}\right).$$

#### Modifying parameters of GNNs based on the value of loss function using Adam optimizer

After defining the model, we expected the model's output to be closer to the true goal by adjusting the model's parameters. The loss function was used to calculate the gap (loss) between predicted output and target, and an optimizer adjusted the model's parameters. In this design, the focal loss [82], was selected as a loss function because it can focus more on class imbalance and misclassified examples. Finally, we used Adam [83], to modify the model's parameters based on the loss value.

### Model validation

The focal loss function was used during the model training to calculate error and optimize the model's parameters using the Adam optimizer. For model optimization, we used a fivefold cross‐validation on the training set to train five models and selected the one which displayed the best classification performance on the testing and external validation sets. The classification performances of the models were evaluated based on specificity (Sp) and sensitivity (Sn), that is, the ability to predict RB correctly and NRB molecules, respectively. The evaluation metrics are calculated using the following equations:

(4) $$\mathrm{Sp}=\frac{\mathrm{TN}}{\mathrm{TN}+\mathrm{FP}},\;\mathrm{Sn}=\frac{\mathrm{TP}}{\mathrm{TP}+\mathrm{FN}}.$$

TN, TP, FN, and FP denote the number of true negatives, true positives, false negatives, and false positives, respectively. In addition, the BA was calculated as the average of sensitivity and specificity. Also, the complement ER of BA was determined. These indices are useful when evaluating the classification performance of a binary classifier. This is particularly relevant when the classes are imbalanced, that is, with an unequal number of molecules in each class [40]. The specificity, sensitivity, BA, and ER are expressed as ratios, not percentages.

### Website architecture and implementation

The website architecture was developed using Django (version 3.1.3), a web back‐end framework for Python (version 3.7). The back‐end system was developed using Python (version 3.6). All data are stored and managed in MariaDB (version 5.5.56). The web interface for the mibPOPdb database was implemented using HTML5, CSS3, JavaScript, and the front‐end framework; Bootstrap (version 4.0). Nucleotide and protein sequences were captured in Biopython and implemented the homolog identification functions by integrating the BLAST (version 2.10.0) package. Clustal Omega (version 1.2.2) and PHYLIP (version 3.695) packages were integrated to implement multiple sequence alignment and phylogenetic tree generation functions. The interactive map and timeline were implemented with the Highcharts Javascript charting library https://www.highcharts.com/products/highcharts/, 2021) and Time Graphics (https://time.graphics/, 2021), respectively.

## AUTHOR CONTRIBUTIONS

**Tanyaradzwa R. Ngara**: Conceptualization, methodology, software, data curation, writing—original draft, writing—review and editing, and visualization. **Peiji Zeng**: Methodology, validation, software, investigation, and data curation. **Houjin Zhang**: Conceptualization, supervision, writing—review and editing, and funding acquisition. All authors have approved the final version of the manuscript.

## CONFLICT OF INTEREST

The authors declare no conflict of interest.

## Supporting information

Supporting information.

Supporting information.

## Data Availability

The mibPOPdb database is freely available at http://mibpop.genome-mining.cn/, and the data sets and standalone code for predicting chemical biodegradability are available at (https://github.com/monsterZeng/MIBPOP/).
