# Supplementary material for: mibPOPdb: An online database for microbial biodegradation of persistent organic pollutants
Source: Imeta. 2022 Aug 17;1(4):e45. doi: 10.1002/imt2.45 (PMC10989864; doi:10.1002/imt2.45)
Supplement: Supplementary file 2 — Supporting information. [file IMT2-1-e45-s001.docx]

**Supplementary materials**

Journal: *iMeta Science*

Title: mibPOPdb: an online database for microbial bioremediation of Persistent Organic Pollutants

Tanyaradzwa R. Ngara^a,#^, Peiji Zeng^a,#^ and Houjin Zhang^a,*^

^a^ *Department of Biotechnology, College of Life Science and Technology, Huazhong University of Science and Technology, MOE Key Laboratory of Molecular Biophysics, Wuhan 430074, China*.

* Corresponding author: Dr. Houjin Zhang,

Address: College of Life Science and Technology, Huazhong University of Science and Technology, MOE Key Laboratory of Molecular Biophysics, Wuhan 430074, China.

Email address: hjzhang@hust.edu.cn

Telephone: +86 27 87793085

^#^These authors contributed equally.

Screening and selection procedures of POP literature used in this study

| **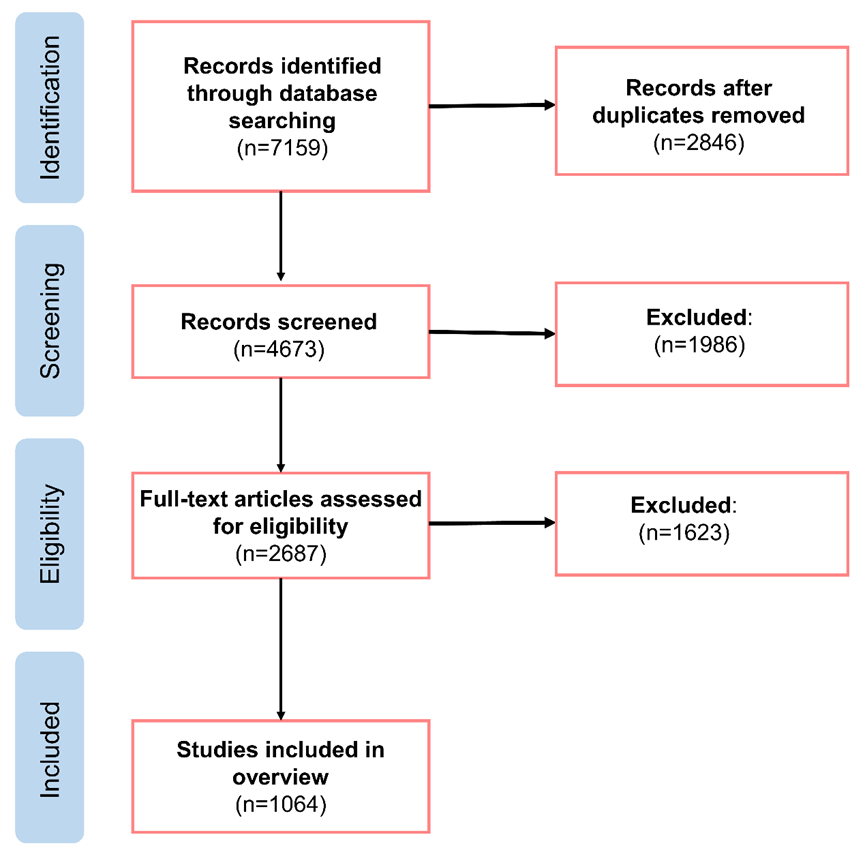**  **Figure S1**. **A flow diagram of study screening and selection procedures.** |
| --- |

**Table S1**: **Ten POPs compounds for which no genes from any organism have been linked to their degradation**.

| **Compound** |
| --- |
| Aldrin |
| Chlordane |
| Chlordecone |
| Dicofol |
| Dieldrin |
| Endrin |
| Heptachlor |
| Hexachlorobutadiene |
| Mirex |
| Toxaphene |

**Table S2: Eligibility criteria**

| Inclusion criteria | Exclusion criteria |
| --- | --- |
| Any original study in which POP-degrading microbes were identified. | Case reports, commentaries, review articles, editorials, letters to the editor |
| Studies in which biodegradation datasets were obtained (i.e., biodegradation rate, primary intermediates, final product, enzyme activity detected, degradation technique, etc.) | Articles which did not include the microbial species responsible for the biodegradation of POPs under study |
| Studies that focused on degradation of POPs regulated under the Stockholm Convention treaty. | Articles which did not identify any novel POP-degrading microbes other than those already mentioned in original literature |
| Biodegradation studies that focused on at least one type of microbe (i.e., Bacteria, Archaea, Fungi, Algae) |  |
| Hand-searched citations from relevant reviews to identify relevant studies that fitted our inclusion criteria |  |

**POP compound entry page**

| **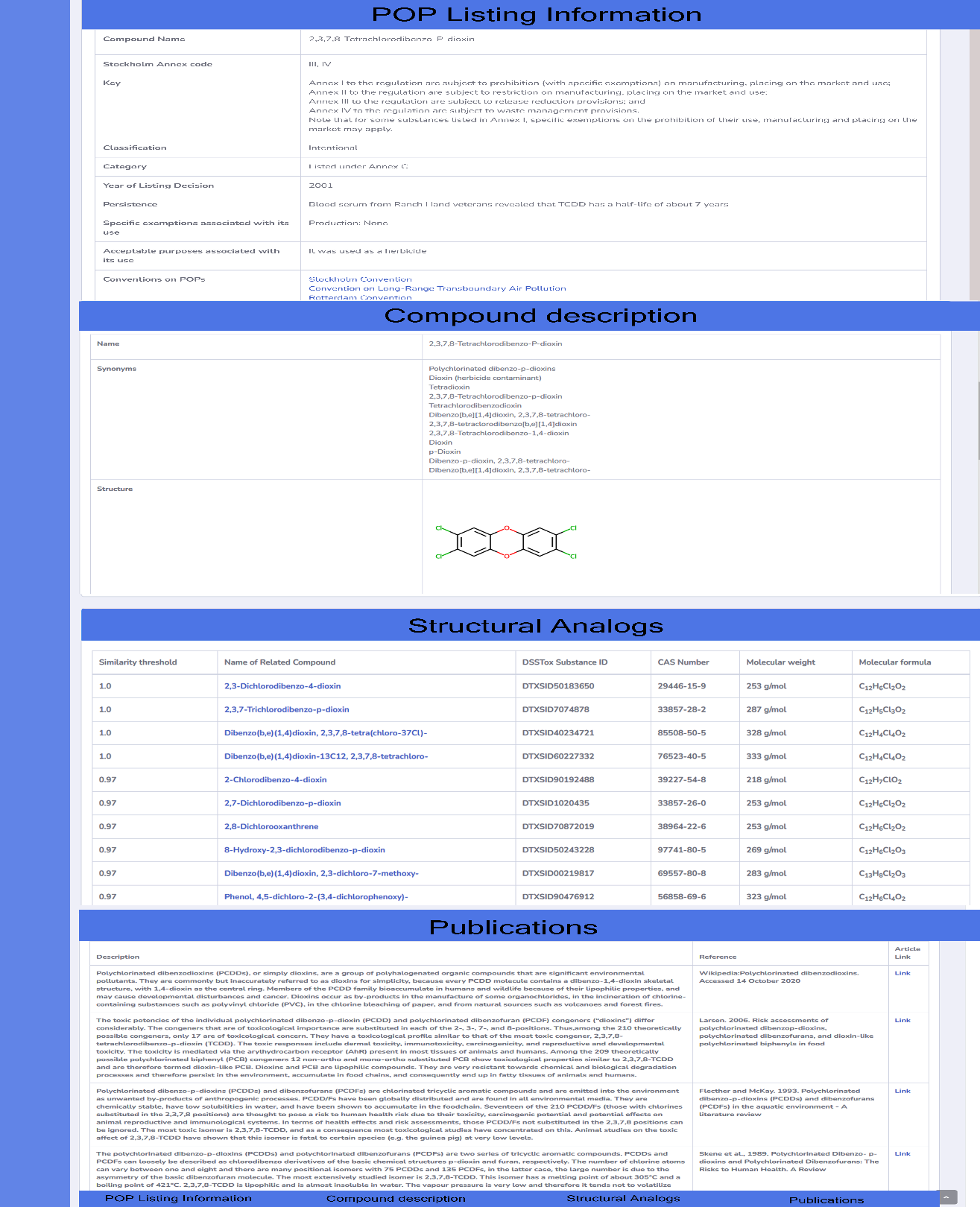**  **Figure S2. Detailed report card page for a POP-compound entry.** It comprises of four sections, i.e., POP listing information, Compound description, Structural analogues, and References to scientific literature information. |
| --- |

**POP degrading microbial strain page**

| **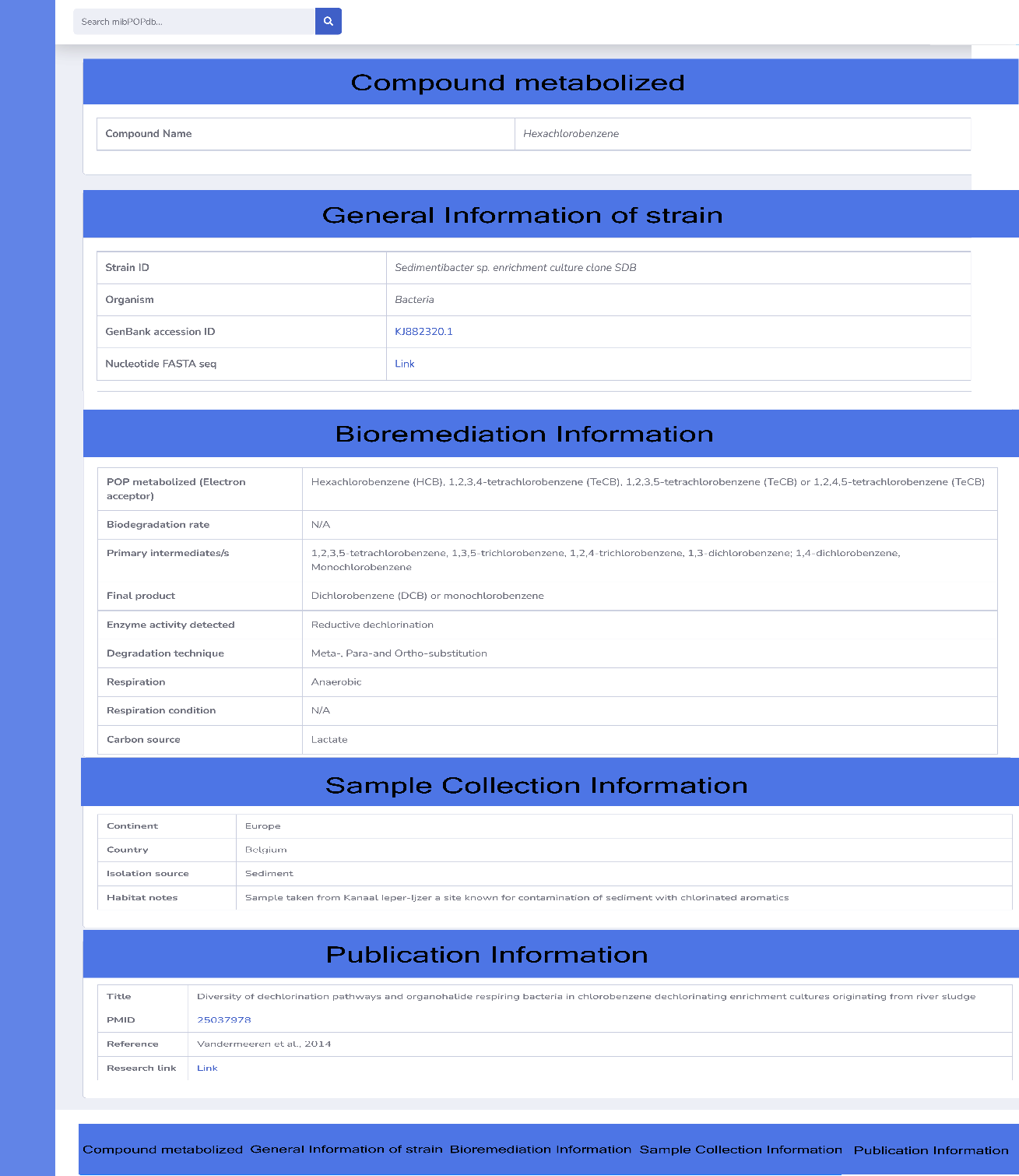Figure S3. Example of a mibPOPdb individual POP degrading microbial strain page.** It comprises five sections, i.e., compound metabolized, strain information, bioremediation information, isolation source, and publication information. For ease of page navigation, the interactive bookmark bar at the bottom of the screen enables users to quickly transverse across the different sections at the click of a button. |
| --- |

**POP biodegradation gene details page**

| **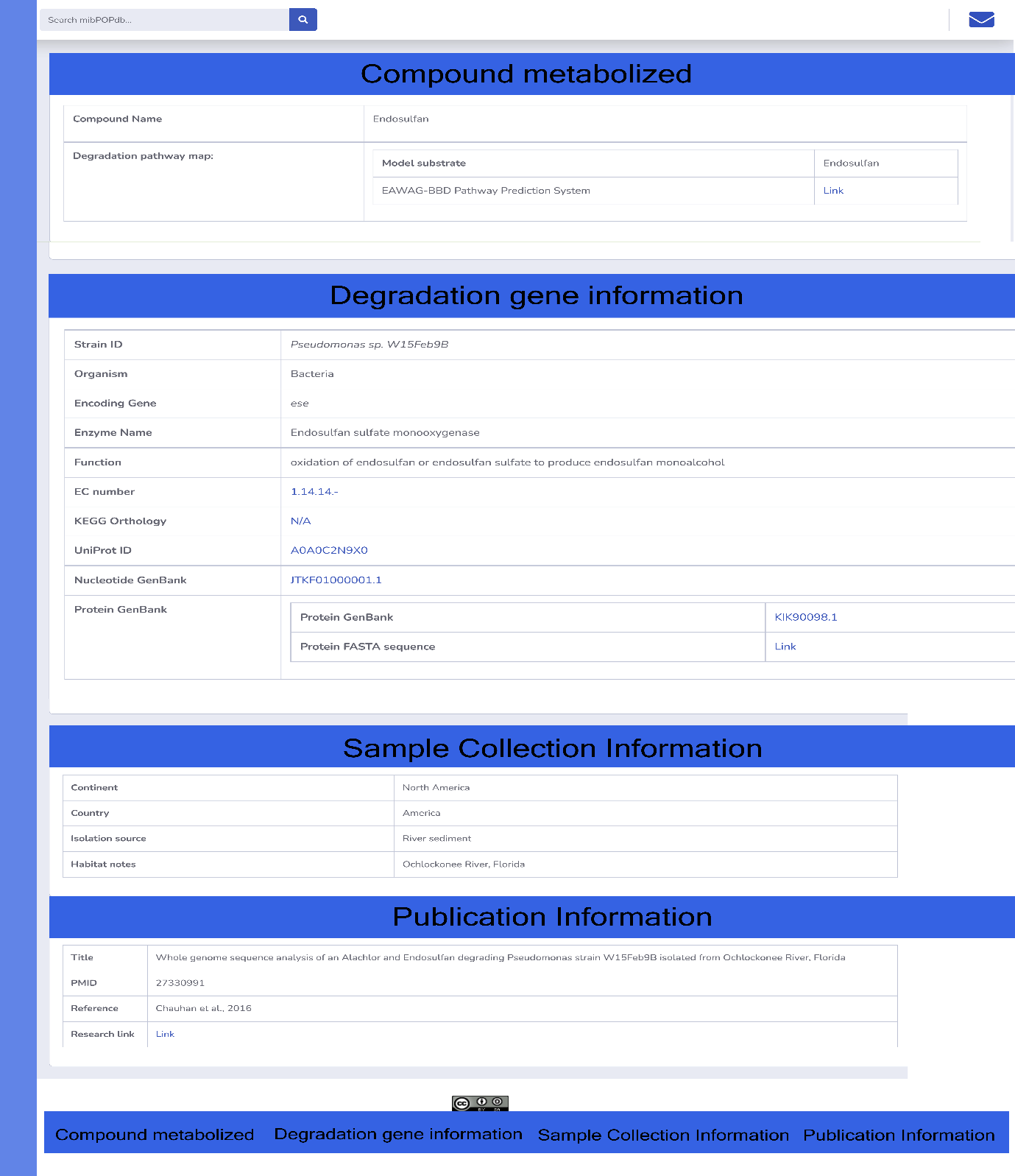Figure S4.** **Screenshot of the POP biodegradation gene details page entry.** (a) Compound metabolized (b) Degradation gene information (c) Sample collection information (d) Publication information. |
| --- |

Intermediate profile page

| **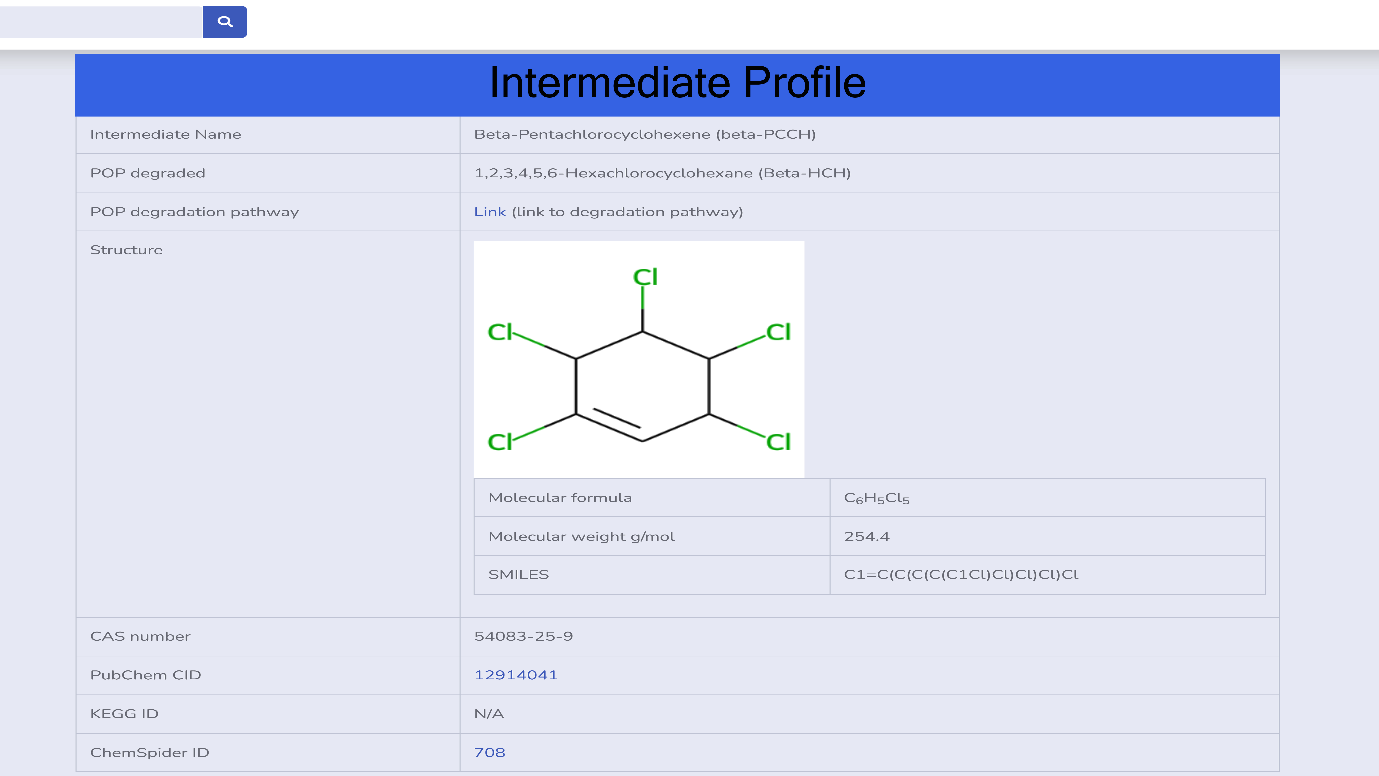Figure S5. Screenshot display of the intermediate compound profile display.** The compound Beta-Pentachlorocyclohexane is an intermediate compound obtained during the biodegradation of 1,2,3,4,5,6-Hexachlorocyclohexane (β-HCH). |
| --- |

Pathway map page

| 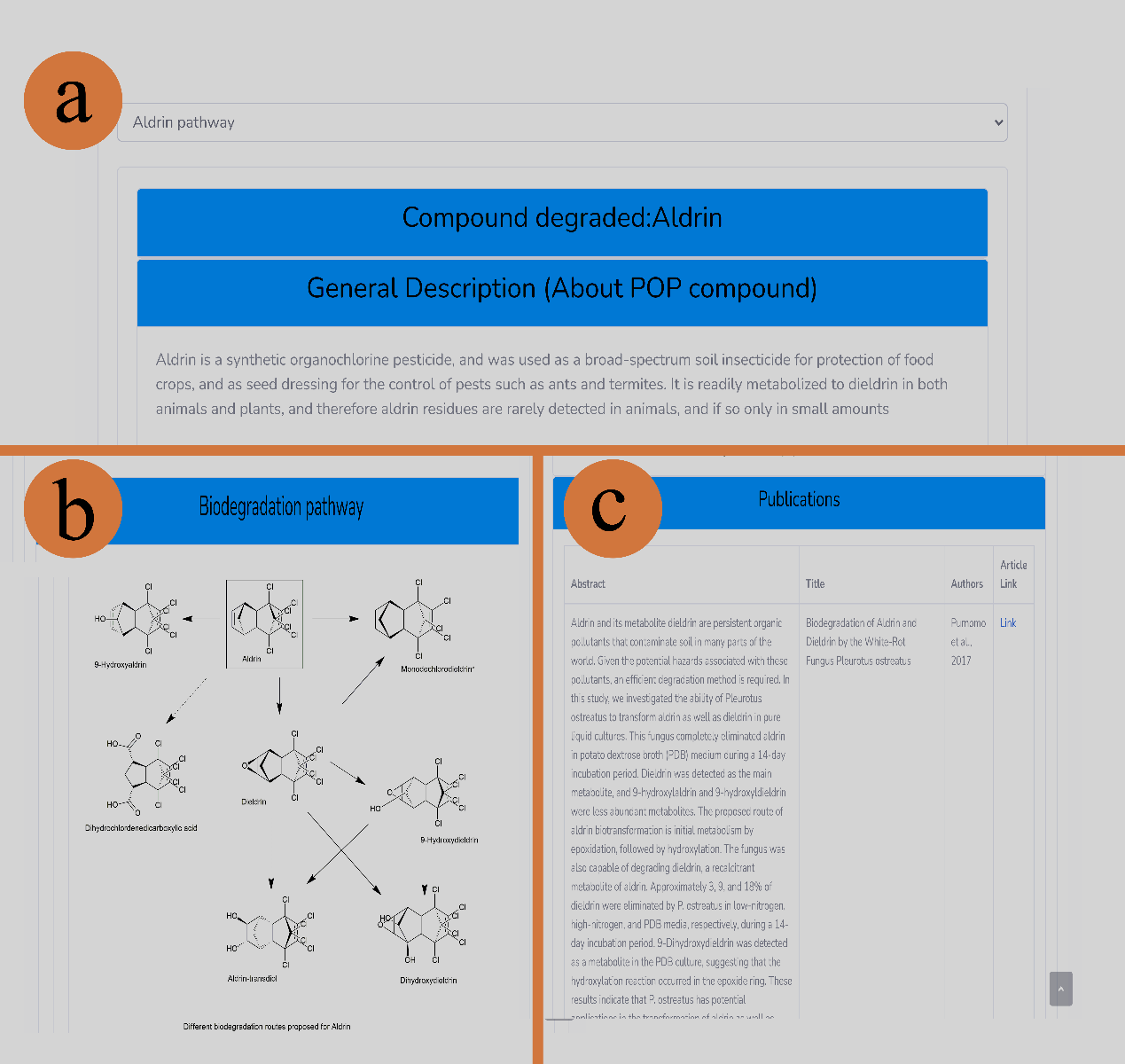  **Figure S6. Screenshot of the pathway map section using the compound Aldrin as an example.** It comprises the information on (a) the compound degraded. (b) biodegradation pathway. (c) references to scientific literature. |
| --- |

**Biodegradative enzyme detailed page.**

| 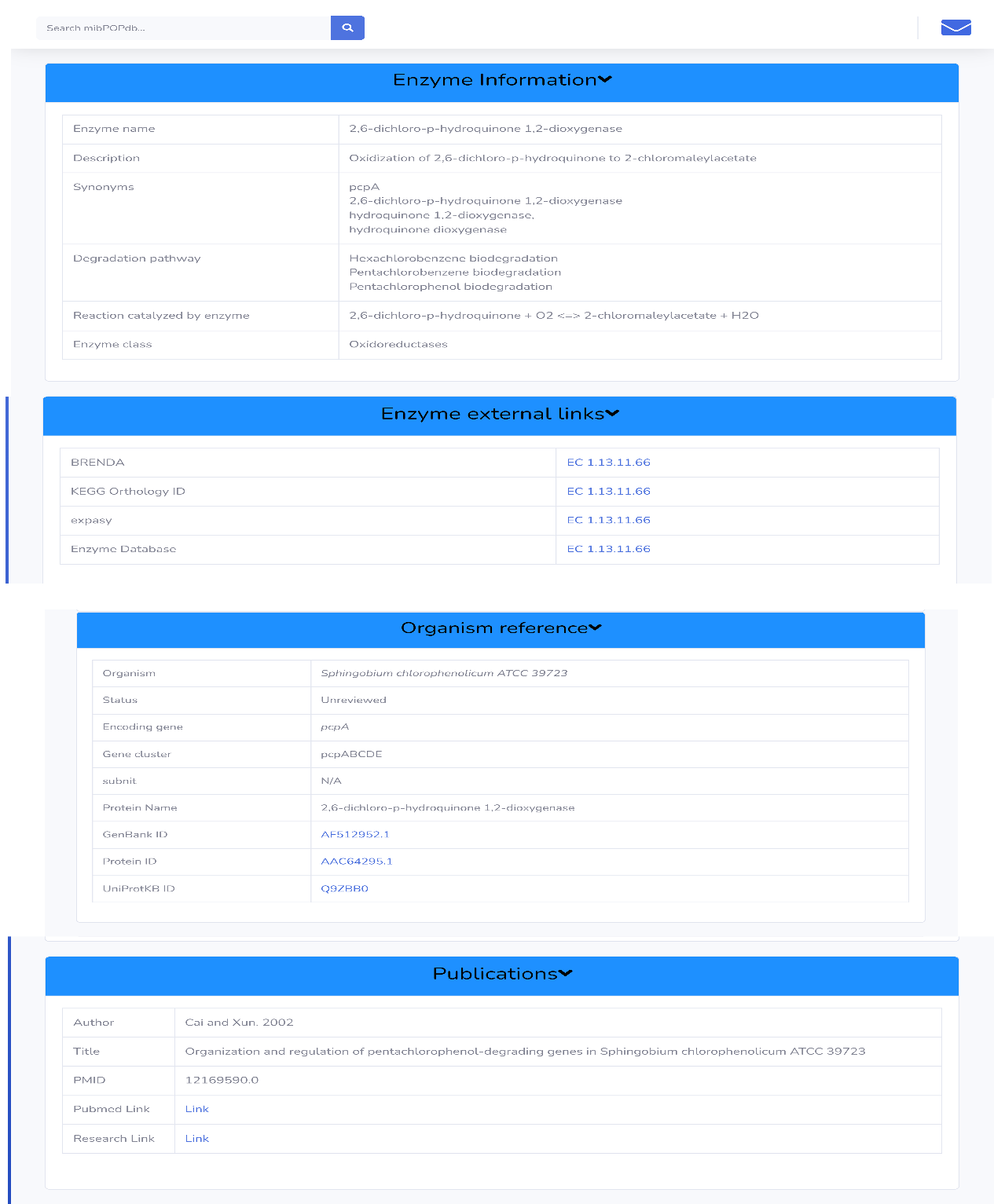  **Figure S7: Screenshot of the biodegradative enzyme details page entry. It comprises the information on (a) the enzyme information. (b) external links. (c) organism reference. (d) publications.** |
| --- |

**Bioinformatics tools incorporated in mibPOPdb Search Sequence Module Interface.**

| 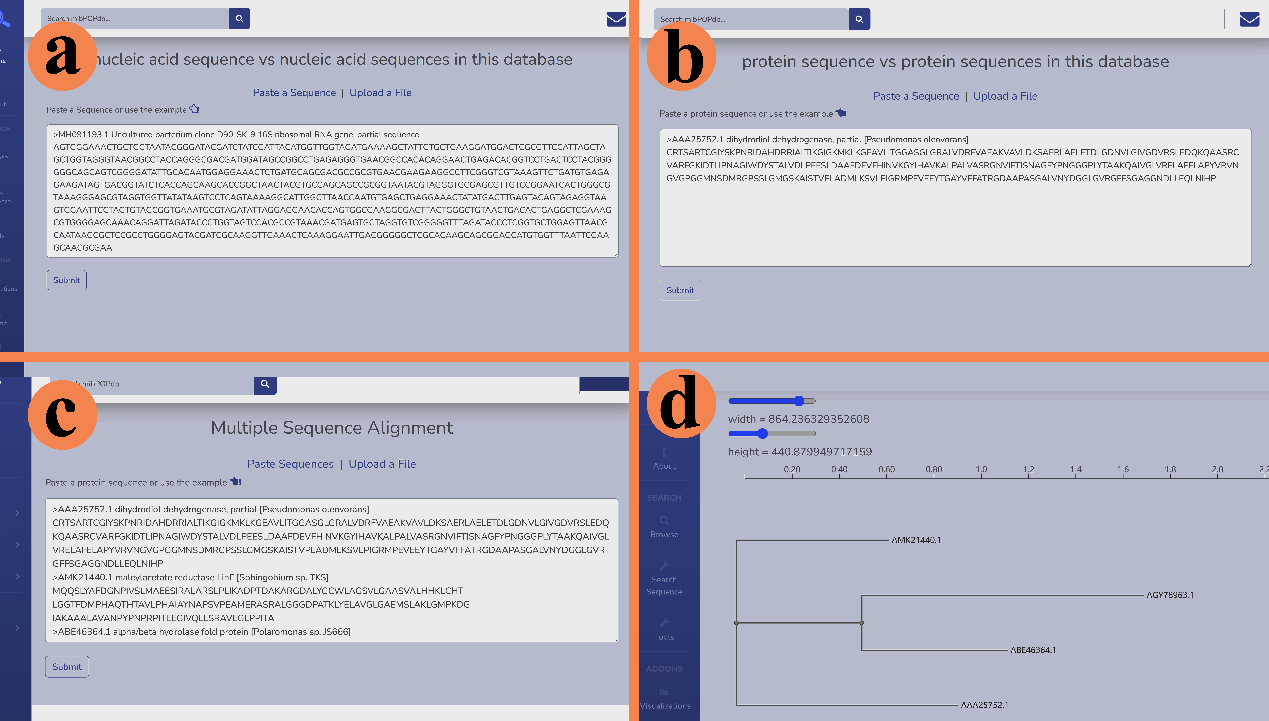  **Figure S8: Screenshots of the search sequence tools.** For (a)BLASTn (b) BLASTp tools, users need to input a query nucleotide or protein sequence respectively in FASTA format to perform local similarity searches. (c) Clustal O for multiple sequence alignment (d) Simple phylogeny, users can submit their aligned sequences to infer homology and analyze phylogenetic relationships between them. |
| --- |

Broad literature searches of the Google Scholar, ScienceDirect, PubMed, Web of Science literature databases. The search strategy is illustrated in Table S2.

**Table S3: The search strategy employed in the PubMed database as an example.**

| **Query** | **Search term** |
| --- | --- |
| **#1** | **"Biodegradation, Environmental"[MeSH]** |
| **#2** | **(Biodegradation, Environmental [Title/Abstract]) OR (Environmental biodegradation[Title/Abstract]) OR (microbial degradation[Title/Abstract]) OR (microbial bioremediation[Title/Abstract])** |
| **#3** | **#1 OR #2** |
| **#4** | **"Specific name of compound under Stockholm Convention or intermediate compounds /metabolism"[MAJR] OR ""Specific name of compound under Stockholm Convention or intermediate compounds /bioremediation"[MAJR] OR "Specific name of compound under Stockholm Convention or intermediate compounds /biometabolism"[MAJR] OR "Specific name of compound under Stockholm Convention or intermediate compounds /catabolism"[MAJR] OR "Specific name of compound under Stockholm Convention or intermediate compounds /decomposition"[MAJR] OR "Specific name of compound under Stockholm Convention or intermediate compounds /biomineralization"[MAJR] OR "Specific name of compound under Stockholm Convention or intermediate compounds /biotransformation"[MAJR]** |
| **#5** | **(Biotransformation[Title/Abstract]) OR (Bioremediation[Title/Abstract]) OR (Decomposition[Title/Abstract]) OR (Catabolism[Title/Abstract]) OR (Biodegradation*[Title/Abstract]) OR (Biomineralization[Title/Abstract]) OR (Xenobiotic/metabolism*[Title/Abstract]) OR (Game[Title/Abstract]) OR (Specific name of compound under Stockholm Convention or intermediate compounds /biodegradation* [Title/Abstract])** |
| **#6** | **#4 OR #5** |
| **#7** | **"** **Specific name of compound under Stockholm Convention or intermediate compounds as Topic"[Mesh] OR (Specific name of compound under Stockholm Convention or intermediate compounds [Publication Type])** |
| **#8** | **(catabolism[Title/Abstract]) OR (degradation[Title/Abstract]) OR (biotransformation[Title/Abstract]) OR (decomposition[Title/Abstract]) OR (bioremediation[Title/Abstract]) OR (biodegradation[Title/Abstract]) OR (metabolism[Title/Abstract])** |
| **#9** | **"Environmental Pollutants as Topic"[Mesh] OR (Environmental Pollutants [Publication Type])** |
| **#10** | **(Specific name of compound under Stockholm Convention or intermediate compounds /biomineralization [Title/Abstract]) OR (Xenobiotic [Title/Abstract]) OR (Pesticides Title/Abstract])** |
| **#11** | **#7 OR #8 OR #9 OR #10** |
| **#12** | **#3 AND #6 AND #11** |

**Deduplication of references in EndNote**

**These are the preferred settings that were employed to interrogate the EndNote records when screening for duplicates. (the process is not entirely automatic and requires visually scanning the records in the later steps)**


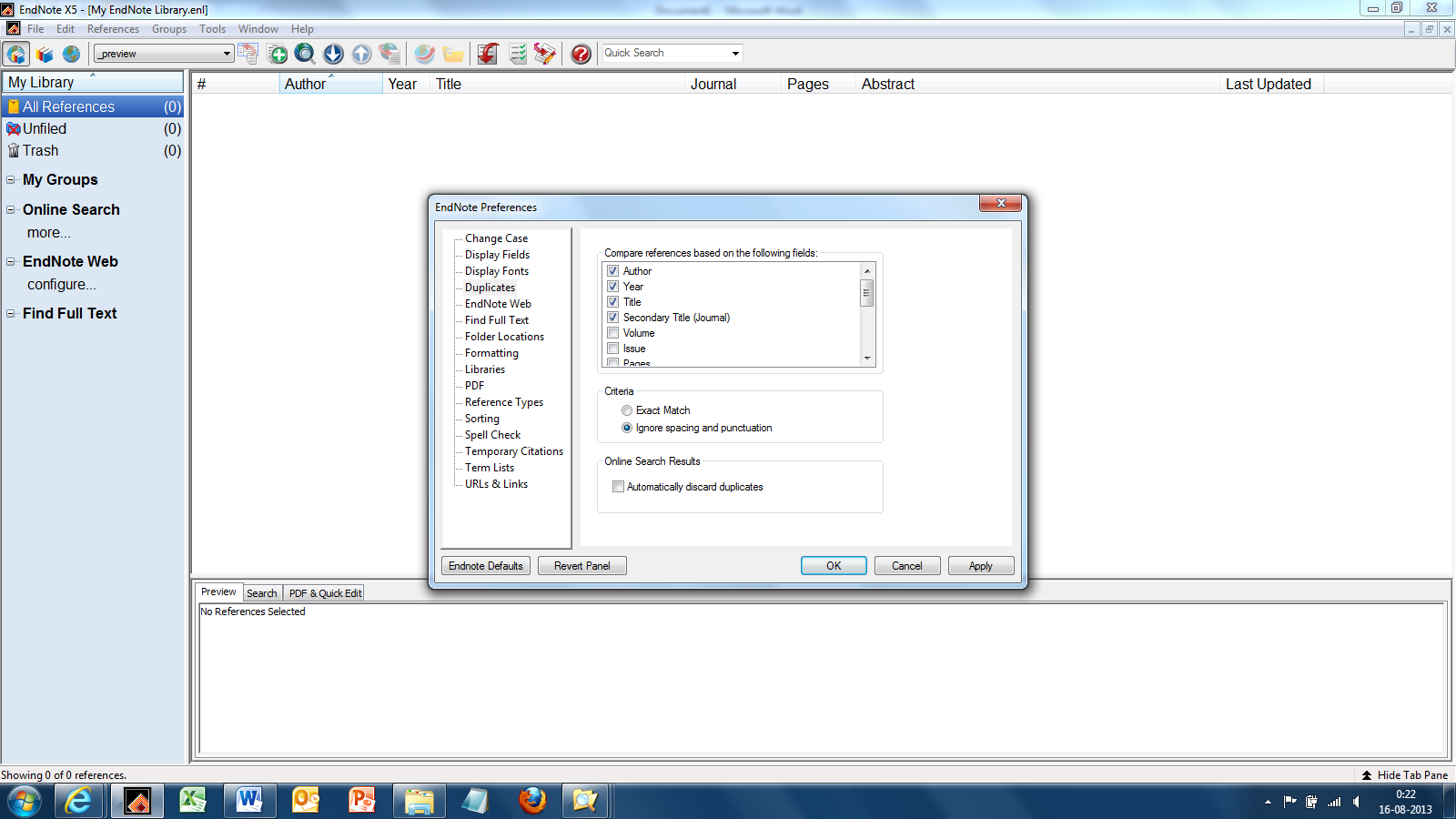
**Edit > preferences > Duplicates**

**Column 1: Author**

**Column 2: Year**

**Column 3: Title**

**Column 4: Journal/Secondary Title**

**Column 5: Volume**

**Column 6: Number**

**Column 7: Pages**

**Column 8: DOI**

**Column 9: Abstract**

**Deduplication steps (search duplicates)**

1. **Highlighted all References in the EndNote library**
2. **Go to References > Find Duplicates**
3. **Click cancel to ignore the suggestion to compare the records individually**
4. **The suggested duplicates remained highlighted and care was taken when clicking so as to not remove the highlighting which will result in the duplicates no longer be identified.**
5. **The duplicates were visually scanned and holding Ctrl-Click were necessary removing highlighting of records that were not actual duplicates.**
6. **All the remaining highlighted references were moved to the trash folder.**
7. **Changed the settings of Edit > Preferences > Duplicates (see the Table S2 below for the fields used in this study) and the steps were repeated.**

**Table S4: Set fields preferences for the deduplication process.**

| **Iteration** | **Set field preferences** | **Compare** |
| --- | --- | --- |
| **1** | **Author; Year; Title; Journal title; Volume; Number; Pages; DOI** | **For blank authors, compare other fields** |
| **2** | **Author; Year; Title; DOI; Pages** | **For blank pages compare Journal title** |
| **3** | **Year; Title; Journal title; Volume; Number; Pages; DOI** | **Author/s** |
| **4** | **Volume; DOI; Journal title** | **Author/s or Title** |
| **5** | **Author; Title** | **Abstract or pages** |

**Table S5 Data fields for POP-degrading microbe entries**

| Compound metabolized | |
| --- | --- |
| Compound Name | Name of the persistent organic pollutant (POPs) toxic chemical/s that adversely affects human health and the environment around the world |
| General Information of strain | |
| Strain ID | Name of the microorganism |
| Organism | An individual form of life (Algae, Archaea, Bacteria and Fungi) |
| GenBank accession ID | GenBank accession number (nucleotide sequence identification number that represents a single, specific sequence in the GenBank database) |
| Nucleotide FASTA seq | A text-based format for representing the nucleotide sequences |
| Bioremediation Information | |
| POP metabolized | Name of the chemical compound degraded |
| Biodegradation rate | The rate at which the chemical compound is transformed or eliminated by the biological action of living organisms |
| Primary intermediates | The chemical substance produced during the conversion of some reactant to a product during the biodegradation process |
| Final product | The final product obtained at the end of the biodegradation process |
| Enzyme activity detected | The presence of a specific enzyme in an organism responsible for the degradation reaction |
| Degradation technique | Routes/behavior of compound biodegradation |
| Respiration | The process in living organisms involving the production of energy |
| Respiration condition | The alternative electron acceptor to oxygen under hypoxic conditions |
| Carbon source | Any molecule which consists of a carbon that a microorganism uses to build biomass |
| Sample Collection Information | |
| Continent | The continent where the original environmental samples were collected |
| Country | The country where the original environmental samples were collected |
| Isolation source | The location where the original environmental samples were collected |
| Habitat notes | The environmental conditions associated with the place an organism makes its home or were sample was collected |
| Publication Information | |
| Title | Expression that indicates the subject of the article |
| PMID | External link to PubMed reference for the document |
| Reference | Author names |
| Research link | External link to the article |

**Table S6 Data fields for POP biodegradation gene dataset**

| Compound metabolized | | | |
| --- | --- | --- | --- |
| Compound Name | |  | |
| Degradation pathway map: | | Model substrate |  |
|  |  | EAWAG-BBD Pathway Prediction System |  |
| Degradation gene information | | | |
| Strain ID | Name of the microorganism | | |
| Organism | An individual form of life (Bacteria and Fungi) | | |
| Encoding Gene | Gene encoding the POP degrading enzyme | | |
| Enzyme Name | Name of the protein identified to degrade the POP or intermediate compound | | |
| Function |  | | |
| EC number | External link to the BRENDA database for the entry | | |
| KEGG Orthology | External link to the KEGG database for the entry | | |
| UniProt ID | External link to the UniProtKB database for the entry | | |
| Nucleotide GenBank | **GenBank ID** | | Nucleotide sequence identification number |
|  | **Nucleotide FASTA sequence** | | A text-based format for representing the nucleotide sequences |
| Protein GenBank | **Protein ID** | | Nucleotide sequence identification number |
|  | **Protein FASTA sequence** | | A text-based format for representing the protein sequences |
| Sample Collection Information | | | |
| Continent | The continent where the original environmental samples were collected | | |
| Country | The country where the original environmental samples were collected | | |
| Isolation source | The location where the original environmental samples were collected | | |
| Habitat notes | The environmental conditions associated with the place an organism makes its home or were sample was collected | | |
| Publication Information | | | |
| Title | Expression that indicates the subject of the article | | |
| PMID | External link to PubMed reference for the document | | |
| Reference | Author names | | |
| Research link | External link to the full version of the article | | |

**Table S7 Data fields for the POP compound page entries**

| POP Listing Information | | | | | | | |
| --- | --- | --- | --- | --- | --- | --- | --- |
| Compound Name | | | Name of the persistent organic pollutant (POP) | | | | |
| Stockholm Annex code | | | Measures taken proposed to be taken by Parties under the treaty (Elimination, Restriction, Unintentional production) | | | | |
| Key | | | Definitions for the different Annexes of Stockholm Convention | | | | |
| Classification | | | Production conditions | | | | |
| Category | | | Type of production (intentional or unintentional) | | | | |
| Year of Listing Decision | | | Year the compound was listed under the SC treaty | | | | |
| Persistence | | |  | | | | |
| Specific exemptions associated with its use | | | Conditions under which the POP compound is allowed to be used under specific restrictions | | | | |
| Acceptable purposes associated with its use | | | Uses associated or that were associated with the POP compound | | | | |
| Conventions on POPs | | | External links to:  Stockholm Convention/ Convention on Long-Range Transboundary Air Pollution/ Rotterdam Convention/ Basel Convention | | | | |
| Compound description | | | | | | | |
| Name | | | Name of the persistent organic pollutant (POP) | | | | |
| Synonyms | | | Other scientific names for the compound | | | | |
| Structure | | | Picture for the structure of the compound | | | | |
| Molecular Formula: | | | **Molecular weight g/mol**: | | **SMILES**: | | |
| CAS Number | | | Chemical Abstracts Service Registry Number of the intermediate compound | | | | |
| European Community (EC) Number | | | Unique identifier | | | | |
| European Chemicals Agency (ECHA) | | | External link to ECHA database | | | | |
| PubChem ID | | | External link to PubChem database | | | | |
| DSSTOX Substance ID | | | External link to DSSTOX database | | | | |
| KEGG | | | External link to KEGG database | | | | |
| ChemSpider | | | External link to ChemSpider database | | | | |
| Structural Analogs | | | | | | | |
| Similarity threshold | **Name of Related Compound** | | **DSSTox Substance ID** | | **CAS Number** | **Molecular weight** | **Molecular formula** |
|  |  | |  | |  |  |  |
| Publications | | | | | | | |
| Description | | **Reference** | | **Article Link** | | | |
| Abstract information | | Author names | | External link to the full version article | | | |

**Table S8 Data fields for the POP intermediate compound entries**

| Intermediate Profile | | |
| --- | --- | --- |
| Intermediate Name | Name of the identified intermediate compound | |
| POP degraded | POP compound degraded by the microbes | |
| POP degradation pathway | Degradation pathway | |
| Structure | Picture of the intermediate compound | |
|  | Molecular formula | Chemical formula which states the number and type of atoms present in a molecule of a substance |
|  | Molecular weight g/mol | Sum of the atomic masses of all atoms in a molecule |
|  | SMILES | A formal language for describing chemical structures |
| CAS Number | Chemical Abstracts Service Registry Number of the intermediate compound | |
| PubChem CID | External link to PubChem database | |
| KEGG ID | External link to KEGG database | |
| ChemSpider ID | External link to ChemSpider database | |

| 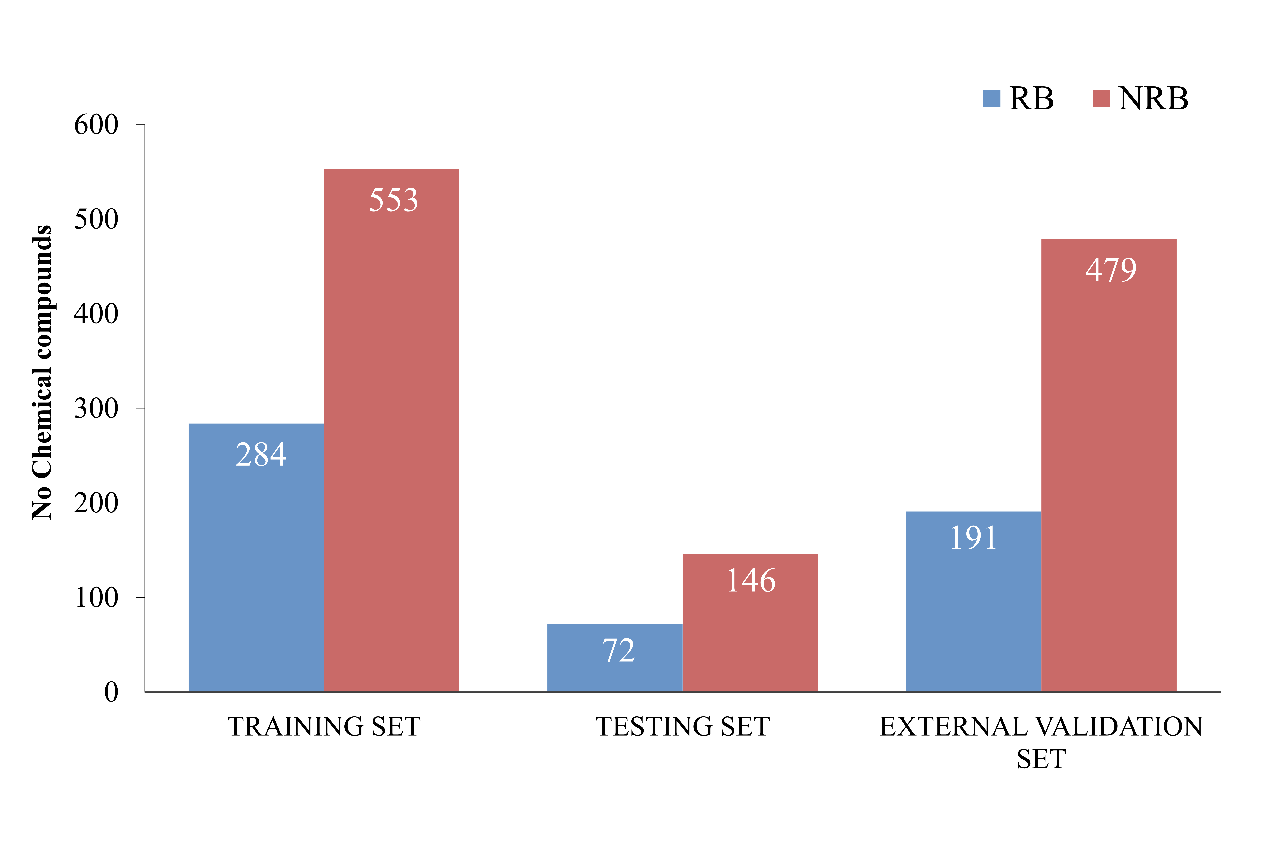  **Figure S9. Distribution of RB and NRB molecules across the three subsets during model training.** |
| --- |

**The biodegradability experimental dataset used to evaluate the GNN model's performance**

**performance**

The feature vector of atom can be used to describe characteristic of atom. Every atom feature vector contains eight types of information, as shown in Table S8.

**Table S9 Featurization for molecules during graph construction**

| Information | Description | Type | Length |
| --- | --- | --- | --- |
| the type of atom | C, N, O, S, F, Si, P, Cl, Br, Mg,Na, Ca, Fe, As, Al, I, B, V, K, Tl, Yb, Sb, Sn, Ag, Pd,Co, Se, Ti, Zn, H, Li, Ge, Cu, Au, Ni, Cd, In, Mn, Zr, Cr, Pt, Hg, Pb | one-hot encoding | 43 |
| degree | the number of connection the node has, the supported possibilities include 0 - 10 | one-hot encoding | 11 |
| implicit Hs | the number of implicit Hs on the atom, the supported possibilities include 0 - 6. | one-hot encoding | 7 |
| Formal charge | Formal charge of the atom, the supported possibilities include -2 - 2. | Numerical | 1 |
| radical electrons | Number of radical electrons of the atom, the supported possibilities include 0 - 4. | Numerical | 1 |
| hybridization | SP, SP2, SP3, SP3D, SP3D2 | one-hot encoding | 5 |
| aromatic | Whether the atom belongs to an aromatic system. the supported possibilities include True or False. | Numerical | 1 |
| total Hs | the number of total Hs on the atom. the supported possibilities include 0 - 4. | one-hot encoding | 5 |

Some characteristics of atom were encoded using one-hot vector, like the type of atom, degree and so on, and some characteristics of atom were encoded using a numerical value. The feature vector of atom concatenates eight types of information and the length is 74.

The architecture of the graph neural network (GNN) layer based on a message-passing neural network (MPNW).

| 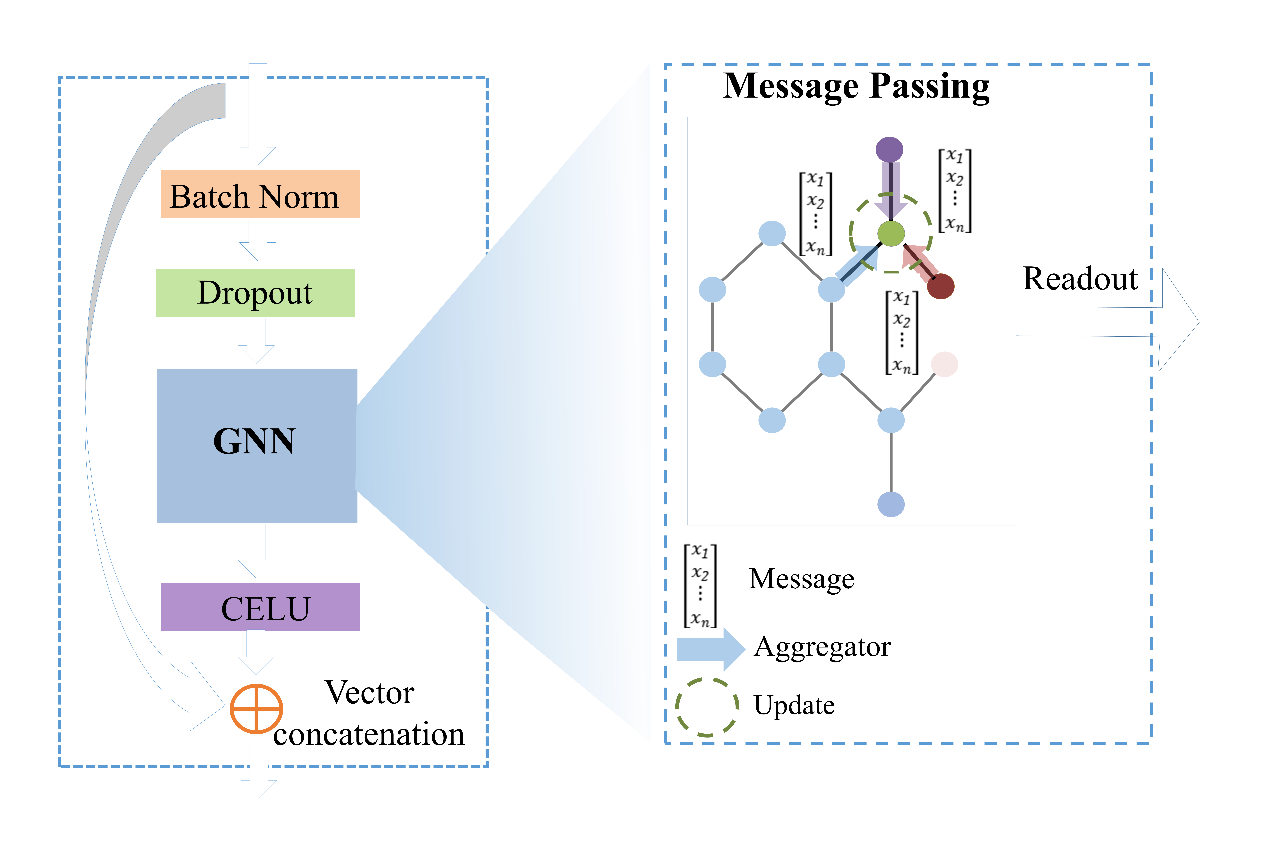  **Figure S10.** **GNN layer based on message passing neural network.** The architecture of the GNN layer is based on a message-passing neural network (MPNW). This layer consists of four functional blocks: batch normalization, dropout, message passing, and CELU. Message passing is at the core of the GNN layer; it extracts local neighborhood information of the node and updates it. Node feature will be copied into two parts; one part will pass through the functional blocks successively. Another part will bypass the functional blocks, and finally, operators will perform vector concatenation on both results. |
| --- |
